# Population-level analysis reveals the widespread occurrence and phenotypic consequence of DNA methylation variation not tagged by genetic variation in maize

**DOI:** 10.1186/s13059-019-1859-0

**Published:** 2019-11-19

**Authors:** Jing Xu, Guo Chen, Peter J. Hermanson, Qiang Xu, Changshuo Sun, Wenqing Chen, Qiuxin Kan, Minqi Li, Peter A. Crisp, Jianbing Yan, Lin Li, Nathan M. Springer, Qing Li

**Affiliations:** 1 National Key Laboratory of Crop Genetic Improvement, Huazhong Agricultural University, Wuhan, 430070 China; 2 Institute of Nuclear and Biological Technology, Xinjiang Academy of Agricultural Sciences, Urumqi, 830091 China; 3 Department of Plant and Microbial Biology, University of Minnesota, St. Paul, MN 55108 USA

**Keywords:** DNA methylation, Gene expression, Phenotypic diversity, Maize

## Abstract

**Background:**

DNA methylation can provide a source of heritable information that is sometimes entirely uncoupled from genetic variation. However, the extent of this uncoupling and the roles of DNA methylation in shaping diversity of both gene expression and phenotypes are hotly debated. Here, we investigate the genetic basis and biological functions of DNA methylation at a population scale in maize.

**Results:**

We perform targeted DNA methylation profiling for a diverse panel of 263 maize inbred genotypes. All genotypes show similar levels of DNA methylation globally, highlighting the importance of DNA methylation in maize development. Nevertheless, we identify more than 16,000 differentially methylated regions (DMRs) that are distributed across the 10 maize chromosomes. Genome-wide association analysis with high-density genetic markers reveals that over 60% of the DMRs are not tagged by SNPs, suggesting the presence of unique information in DMRs. Strong associations between DMRs and the expression of many genes are identified in both the leaf and kernel tissues, pointing to the biological significance of methylation variation. Association analysis with 986 metabolic traits suggests that DNA methylation is associated with phenotypic variation of 156 traits. There are some traits that only show significant associations with DMRs and not with SNPs.

**Conclusions:**

These results suggest that DNA methylation can provide unique information to explain phenotypic variation in maize.

## Background

DNA methylation is the most studied chromatin modification in many plant species. DNA methylation has important roles in maintaining genome integrity and may also influence plant development and environmental responses, especially in species with complex genomes [1–6]. DNA methylation occurs in three sequence contexts in plants, CG, CHG, and CHH (H = A, C, or T), each of which is maintained by different pathways. CG is maintained by DNA METHYLTRANSFERASE 1 (MET1), CHG by CHROMOMETHYLASE 3 (CMT3), and CHH by RNA directed DNA Methylation (RdDM) as well as CHROMOMETHYLASE 2 (CMT2) [2, 7, 8].

DNA methylation often varies across different individuals of the same species [9–13]. This can include spontaneous epimutations [9–11] as well as differences among genetically distinct varieties. Natural variation for DNA methylation includes examples in which genetic changes such as transposon insertions or rearrangements cause the change in methylation (obligatory epialleles) [14, 15]. There can also be more complex examples in which a genetic change results in a facilitated epiallele [14]. Other examples of natural variation for DNA methylation can reflect pure epigenetic variation that occurs in the absence of any causative genetic changes [1, 14]. The association between DNA methylation and genetic variation has been reported at a genome-wide scale in *Arabidopsis* [12, 16, 17]. These studies suggest that some examples of variation for DNA methylation are due to genetic changes while others are not. In maize, a species with a larger genome and much higher transposon content, there is also evidence of both genetic-dependent and genetic-independent DNA methylation [18, 19]. The previous studies in maize were not able to detect context-specific DNA methylation patterns, which may have different functions and stabilities.

DNA methylation can result in phenotypic changes, most likely through influencing gene expression. DNA methylation within a gene can affect splicing of pre-mRNAs [20, 21]. DNA methylation outside gene, especially those within promoter regions, has been suggested to influence gene expression levels [22]. Several studies in plant species suggest that the number of genes whose expression is affected by DNA methylation is limited, at an order of hundreds, and the genes whose expression is affected by DNA methylation usually have specific properties [13, 23]. These genes generally show qualitative changes (on versus off) in expression among different genotypes [13]. However, these studies assayed gene expression in one tissue. It is possible that more genes for which expression are associated with DNA methylation can be identified if more tissues were assayed, as has been found for genetic variation [24–27].

In this study, we investigated the genetic basis and biological consequences of natural variation in DNA methylation among diverse inbred lines in maize. We found evidence for many examples of differentially methylated regions (DMRs) that contain information that is not fully captured using SNPs. We showed that variation in DNA methylation is associated with variation in gene expression, and this association is dependent upon sequence contexts as well as the position of DMR relative to gene transcriptional start site. Furthermore, we showed that variation in DNA methylation is associated with phenotypic variation in maize and can explain a portion of the heritability of some metabolic traits.

## Results

### Extensive variation in DNA methylation among maize inbred lines

To explore natural variation in DNA methylation, we profiled DNA methylation across a panel of 263 diverse maize inbred lines using a capture-based method [28]. This method allows single-base resolution of DNA methylation across a large population at a common set of loci with high coverage. The capture space includes over 20,000 regions, covering 15 Mb of the maize genome [29]. These regions were selected based on our previous work of DNA methylation in maize and include regions that vary in DNA methylation across three maize lines or five maize tissues, mCHH islands, and promoters of genes that are potentially silenced by DNA methylation [13, 30]. The maize inbreds utilized in this study represent a wide range of diversity, including lines that are adapted to tropical/semitropical regions or temperate regions. It includes the inbred B73 used to make the maize reference genome as well as inbreds widely used in past and present breeding schemes (Additional file 2: Table S1). We obtained a total of ~ 2.3 billion 2 × 125 paired reads, with an average of ~ 8.7 million paired reads for each sample (Additional file 2: Table S1). These reads were mapped to the B73 genome [31] at a mapping rate from 16 to 70%, corresponding to an average of ~ 3.8 million mapped reads for each sample. At this mapping rate, we have a total of ~ 7 Mb of regions with ≥ 2-fold coverage in > 60% of the lines, allowing us to identify regions with variable DNA methylation at a population scale in maize.

**Fig. 1:**
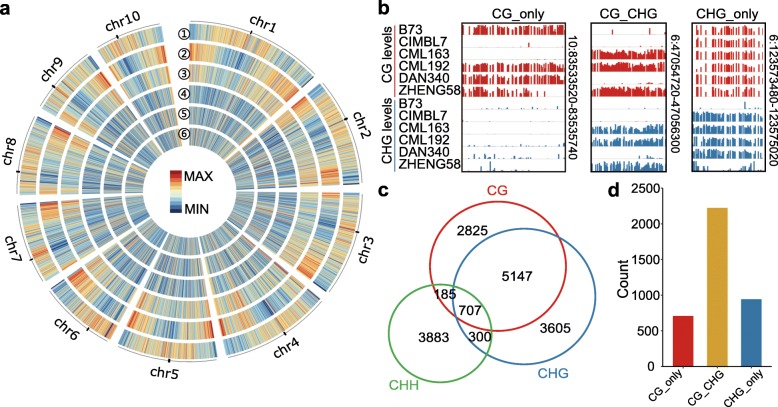
Natural variation of DNA methylation among maize inbred lines. **a** The distribution of DMRs throughout maize chromosomes and the relationship with other genomic features is visualized. From the outer ring to the inner ring (1–6) are TEs, genes, regions with capture probes, CG DMRs, CHG DMRs, and CHH DMRs. Density of each type was calculated based on 1 Mb windows, and the centromeres are represented by black blocks. **b** Examples of context-specific DMRs. Each track represents different inbred lines. **c** Overlaps between CG, CHG, and CHH DMRs. **d** Total number of context-specific DMRs

DMRs were identified for each of the three sequence contexts. In total, we identified 8864, 9759, and 5075 DMRs for CG, CHG, and CHH respectively. To support the reliability of the capture-based assay of DNA methylation, we compared DMRs that are identified using this method with the DMRs that are identified based on whole genome bisulfite sequencing (WGBS) [13]. We took advantage of B73 and Mo17 for which DNA methylation levels have been assayed using both methods. As expected, the DMRs that are identified using the capture-based method are well supported by WGBS data (Additional file 1: Figure S1). The DMRs were distributed along the 10 maize chromosomes. Many of the DMRs were located in regions annotated as genes, corresponding well with the distribution of capture probes (Fig. 1a, Additional file 1: Figure S2). The size of DMRs is quite similar for all three sequence contexts, with the majority between 60 bp and 1000 bp and a median of 200 bp (Additional file 1: Figure S2a). This suggests that variation in DNA methylation is usually confined to a region corresponding to one or several nucleosomes. The analysis of the minor epiallele frequency (MEF) suggests that the lowly methylated state is often the rare epiallele for CG and CHG DMRs while CHH DMRs often exhibit higher methylated levels as the rare epiallele (Additional file 1: Figure S2b). The distribution of CG and CHG DMRs relative to genes and TEs is similar to the distribution of all assayed regions. The CHH DMRs are depleted within genes (Additional file 1: Figure S2c). Prior studies in *Arabidopsis* have identified natural variation in DNA methylation components such as VIM or CMT2 [17, 32, 33] that influence genome-wide methylation levels or patterns in some ecotypes, but we did not find any evidence for this in maize.

Though we identified DMRs separately for each context, there are many loci at which they can co-vary (Fig. 1b). In fact, there is substantial redundancy for DMRs for different contexts, especially for CG and CHG DMRs (Fig. 1c, d). We thus combined CG and CHG DMRs, and identified a set of regions that show variations in both contexts or only in CG or CHG context. A stringent set of criteria were applied (the “Methods” section) to identify 708 DMRs that vary only in CG context (CG_only), 944 DMRs that vary only in CHG context (CHG_only), and 2223 DMRs that vary in both CG and CHG contexts (CG_CHG). The relative frequency of context-specific DMRs within the captured loci is quite similar to the genome-wide patterns [13]. The size range of the context-specific DMRs is quite similar, but the overlap with genomic features is distinct (Additional file 1: Figure S2d, e).

### DMRs can differentiate maize subgroups

**Fig. 2:**
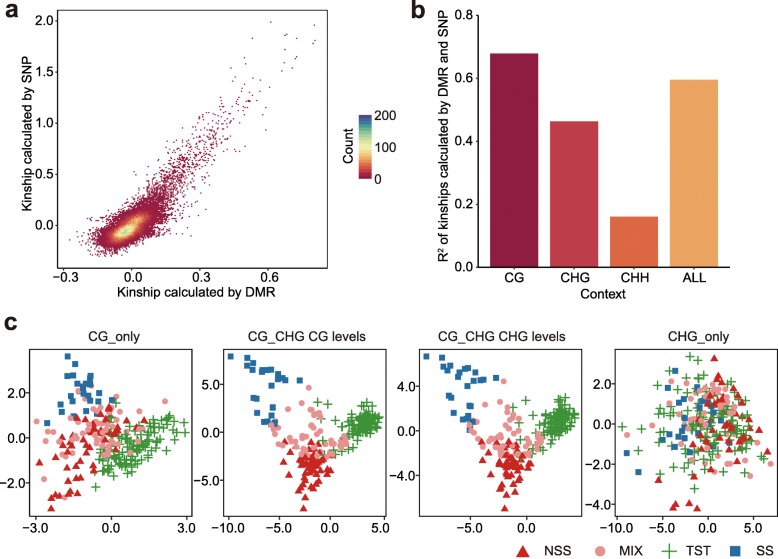
Comparison of genetic distance calculated using either SNPs or DNA methylation. **a** The correlation of kinships between any two lines calculated by SNPs or DNA methylation in CG context. The color reflects the density of points. **b** The squared Pearson correlation coefficient (*R*^*2*^) between the two kinships calculated using either SNPs or the indicated DNA methylation. ALL means that the kinship was calculated using all three contexts. **c** PCA plots of context-specific DMRs. The color and shape of symbols reflect different subgroups of maize that are determined by SNPs. SS, stiff stalk; NSS, non-stiff stalk; TST, tropical or semi-tropical

We explored whether variation in DNA methylation accurately reflects genetic relationships among different inbred lines. Individual relatedness that is computed using either SNP or DNA methylation levels is highly correlated (Fig. 2a). Interestingly, the correlation is substantially higher for CG methylation at CG DMRs than for CHG (CHG DMRs) and CHH (CHH DMRs) methylation (Fig. 2b), suggesting that CG methylation is more stable. Principle component analysis (PCA) performed using CG, CHG, or CHH methylation levels from DMRs can separate inbreds into different subgroups, which agree well with classifications based on SNPs (Additional file 1: Figure S3a). The ability to differentiate population subgroups is highest for CG methylation (Additional file 1: Figure S3a). We then asked whether variable levels of CG (or CHG) methylation from CG_only (or CHG_only) DMRs have similar power as that from CG_CHG DMRs to differentiate populations (Fig. 2c). Interestingly, the CG_only and CHG_only DMRs show less correlation with genetic distance than CG_CHG DMRs. The differentiation of population subgroups using DMRs suggests the presence of subgroup-specific DMRs. Indeed, an analysis of variance analysis (ANOVA) suggests that there are numerous DMRs that show high or low methylation in the majority of lines of a subgroup, though no DMRs that have exclusively low or high methylation in one subgroup were identified (Additional file 1: Figure S4).

### Genome-wide association analysis to dissect the genetic basis of DMRs

**Fig. 3:**
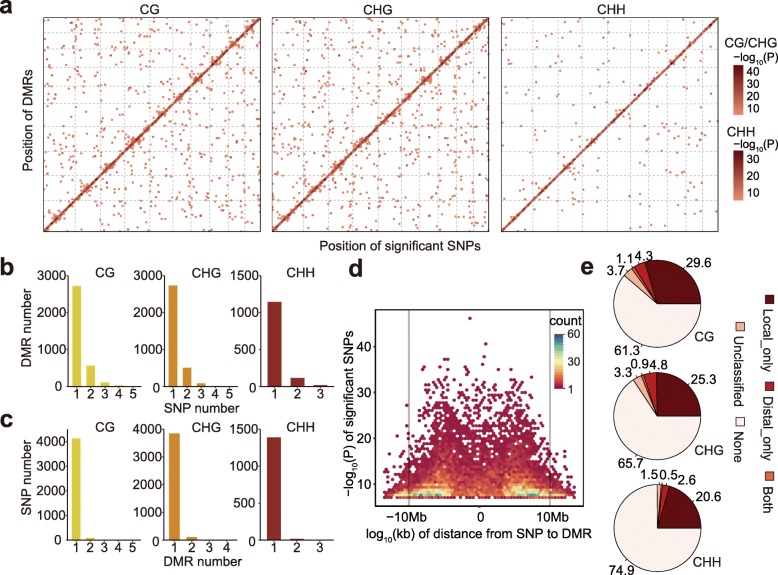
The genetic basis of DMRs. **a** The genomic distribution of DMRs and their associated SNPs is shown. The *x*-axis indicates the genomic positions of the significant SNPs, while the *y*-axis shows the genomic positions of the corresponding DMRs. The color of each point reflects the *P* value. **b** Distribution of the number of significant SNPs per DMR. **c** Distribution of the number of significantly associated DMRs for each SNP. **d** The distance between DMR and the significant SNP. The *y*-axis represents the *P* value, and the color reflects the density of associated SNP-DMR pairs. **e** Summary of the genetic basis for CG, CHG, and CHH DMRs

DNA methylation could be associated with nearby genetic variation either due to the causal action of the genetic variation upon methylation levels or due to linkage disequilibrium (LD) between highly stable DMRs and nearby genetic changes. To investigate to what extent DMRs are tagged by SNPs and whether there are differences among different types of DMRs, we performed genome-wide association analysis (GWAS) with ~ 1 million high-quality SNPs using a mixed linear model that controls for both population structure and individual relatedness. We identified a total of 4336, 4096, and 1426 associations for CG, CHG, and CHH DMRs (Fig. 3a and Additional file 2: Table S2) respectively at a genome-wide significance level of 5.15 × 10^−8^ (Bonferroni-corrected *P* value of multiple testing). Of the DMRs with associations, most shows significant association with only one SNP (Fig. 3b). Similarly, most of the SNPs associate with only one DMR (Fig. 3c). A close examination of the distance between the DMR and the associated SNPs shows that the distance is usually within 10 Mb of each other (Fig. 3d). Thus, the SNPs were defined as local if the associated DMR is within 10 Mb and were defined as distal if on a different chromosome. In agreement with the observation that no maize genotypes had global changes in DNA methylation in the association panel, no strong distal hotspots were identified (Fig. 3a). This suggests that the major methylation machinery was not compromised in the panel of inbreds used for this study.

Of the three sequence contexts, CG is most likely associated with SNPs either by local or by distal, and rarely by both (Fig. 3e). CHH DMRs are the least to show associations with SNPs. Importantly, > 60% of the DMRs do not have a significant association with any SNPs for each of the three sequence contexts (Fig. 3e), suggesting that these DMRs may represent unique information that is not captured by SNPs and therefore would not be represented in typical GWAS analyses. In some cases, the DMRs could arise from structural variants that may not be effectively tagged by SNPs. The set of structural variants identified by Yang et al. [34] were used for an association analysis with the DMRs (Additional file 1: Figure S5). This identified an additional 0.3–1% of the DMRs that do not have significant associations with SNPs that can be associated with a structural variant. Overall, these analyses suggest that a substantial portion of DMRs are not effectively captured by SNPs or structural variants.

### Variation in chromatin and genetic control of context-specific DMRs

**Fig. 4:**
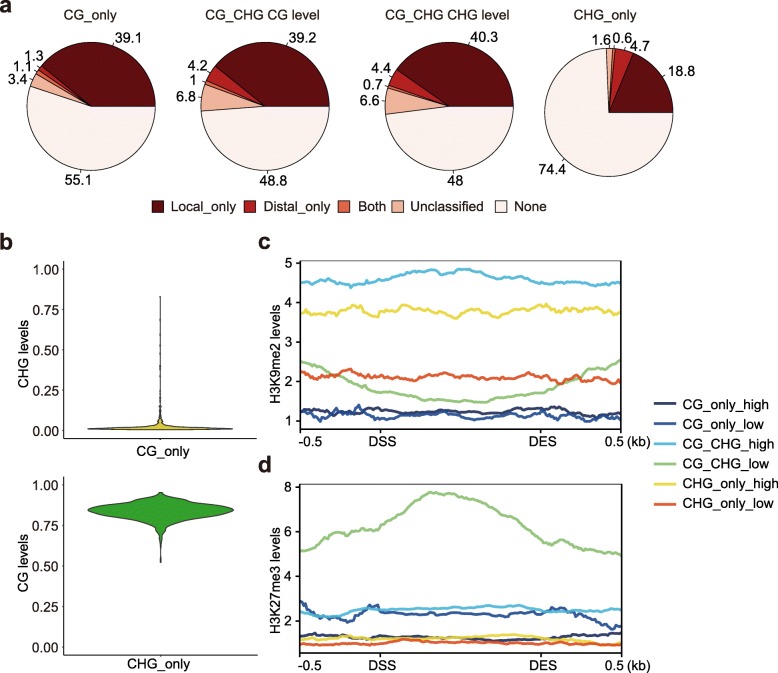
Regulation of context-specific DMRs. **a** Summary of the genetic basis for context-specific DMRs. **b** CHG methylation levels in CG_only DMRs and CG methylation levels in CHG_only DMRs. **c** H3K9me2 metaprofile around context-specific DMRs. **d** H3K27me3 metaprofile around context-specific DMRs

There is variation for the frequency of DMRs for different sequence contexts that exhibit significant association with SNPs. The proportion of DMRs with significantly associated SNPs is lower for CG_only (45%) and CHG_only (26%) DMRs than that for CG_CHG DMRs (51%) (Fig. 4a). This suggests that CG_only and CHG_only DMRs are less stable than CG_CHG DMRs, consistent with the above observation that CG_CHG DMRs have better power in differentiating maize subgroups. To investigate the properties of context-specific DMRs, we looked at the methylation levels of the other contexts in CG_only and CHG_only DMRs. For most of the CG_only DMRs, the CHG levels are uniformly low across all genotypes (Fig. 4b), suggesting that these regions are not targets of *Zmet2/Zmet5*, which maintains CHG methylation. In contrast, most of the CHG_only DMRs have high levels of CG methylation in all genotypes (Fig. 4b), suggesting that these regions are consistent targets of CG methylation maintaining enzyme, MET1. There is also variation in the chromatin features of context-specific DMRs (Fig. 4c, d). CG_only DMRs tend to have low levels of both H3K9me2 and H3K27me3 marks. This suggests that CG_only DMRs are less likely to occur in regions with repressive chromatin environments, in agreement with the findings that they are enriched in genic regions and likely represent differential gene body methylation (Additional file 1: Figure S2). CHG_only DMRs tend to have higher H3K9me2. The high levels of H3K9me2 were only observed in the subset of CHG_only DMRs that have high DNA methylation in the B73 where H3K9me2 data was collected, not in the DMRs where DNA methylation is low in B73. This agrees with the fact that CHG methylation and H3K9me2 form a self-reinforcing loop [35]. In contrast to CG_only and CHG_only DMRs, CG_CHG DMRs have high levels of both H3K9me2 and H3K27me3. Interestingly, the high levels of H3K9me2 occur in DMRs where DNA methylation is high in B73 (the genotype where H3K9me2 and H3K27me3 were collected), while the high levels of H3K27me3 occur in DMRs where DNA methylation is low in B73 (Fig. 4c, d). This agrees with the reports that DNA methylation and H3K27me3 are generally repulsive [36].

### DMRs are associated with natural variation in gene expression

**Fig. 5:**
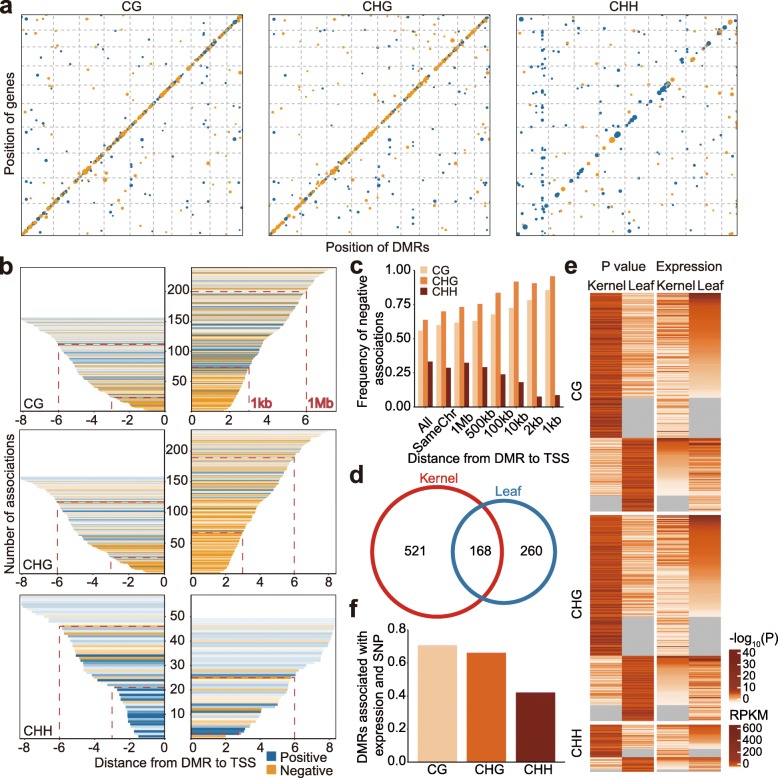
Associations between DMRs and gene expression. **a** The genomic distribution of genes and their associated DMRs. The *x*-axis indicates the genomic positions of DMRs, while the *y*-axis is the genomic positions of corresponding genes. The color reflects the direction of association between gene expression and DMRs, with blue for positive associations and orange for negative associations. Note there are more orange dots (negative associations) in the plots of CG and CHG methylation, and more blue dots (positive associations) in the plot of CHH methylation. **b** The distance between the DMR and the gene transcription start site (TSS) is evaluated for DMR-gene expression associations. The *x*-axis is the log_10_ (downstream) or − log_10_ (upstream) value of the distance from DMR to the TSS, while the *y*-axis is the number of associations. The color indicates the direction of associations with darker shades representing lower *P* value. **c** Summary plot for the proportion of negative associations for DMRs with varying distance to gene TSS. Note the closer the DMR to gene, the more negative associations for CG and CHG methylation and the more positive associations for CHH methylation. **d** Comparison between leaf and kernel expression data using associations within 1 Mb from DMR to TSS. **e** The expression levels of genes in leaf (kernel) which only show significant associations in kernel (leaf) tissue. The left plot shows *P* value of significant gene-DMR associations, and the right plot shows levels of gene expression. Gray color means that the data are not available because of low/no expression of the gene in that tissue. **f** The proportion of DMRs associated with gene expression that also have a significant association with SNPs

To investigate the association between DNA methylation and gene expression, we performed GWAS using gene expression as the dependent variable and DNA methylation as the independent variable. The expression data was from kernels 15 days after pollination. At *P* < 1 × 10^−6^ (Bonferroni’s correction for multiple test, 0.01/*N*, *N* is the number of DMRs), 1389 significant associations between expression and methylation levels were detected, with CG, CHG, and CHH DMRs having 538, 562, and 289 associations, respectively (Fig. 5a, Additional file 2: Table S3). The majority of the significantly associated DMRs were located on the same chromosome as the genes, and the distance between the DMR and the gene is usually less than 1 Mb (Additional file 1: Figure S6a). The majority of DMRs are associated with only one gene, arguing against the presence of distal hotspots (Fig. 5a).

There are more negative associations for CG and CHG DMRs (Fig. 5a–c). In contrast, CHH DMRs tend to be positively associated with gene expression. These trends become stronger the closer the DMR is to the transcription start site (TSS), especially for DMRs within 1 kb of TSS (Fig. 5b, c). Many of the negative associations for CG and CHG were located downstream of the TSS (Fig. 5b), suggesting that high CG and CHG methylation within the gene body are likely to associate with reduced expression. It is observed that all three sequence contexts, CG, CHG, and CHH, from the same DMR can associate with expression of the same gene though the direction of association could be different (Additional file 1: Figure S6b). Interestingly, CG_CHG DMRs are ~ 3 times more likely to be associated with gene expression compared with CG_only and CHG_only DMRs (Additional file 1: Figure S6c). The CHG methylation from either CG_CHG DMRs or CHG_only DMRs tend to be negatively associated with gene expression, while CG methylation shows negative associations when it co-varies with CHG methylation but shows positive associations (> 75% cases) when in CG_only DMRs (Additional file 1: Figure S6d). Thus, the association between gene expression and DNA methylation is dependent upon sequence context.

To assess how DMRs are associated with gene expression in different tissues, we explored the association between DMRs and gene expression in a second expression dataset derived from leaf tissue, and compared with the associations from the developing kernels. We identified a total of 1096 associations in the leaf tissue, compared to 1389 in the kernel tissue. There are 689 and 428 DMR-gene associations which are located within 1 Mb of each other in kernel and leaf, respectively, including 168 associations that were identified in both tissues (Fig. 5d). The shared associations include 74, 79, and 15 for CG, CHG, and CHH DMRs respectively. While some DMRs show common associations, there are many DMRs that only associate with gene expression in one of the tissues (Fig. 5d). A close examination of the data revealed that 3.7–12.5% of tissue-specific associations are significant in another tissue if less stringent criteria were applied, and 17~29% were due to tissue-specific expression patterns of genes (Fig. 5e). For the remaining gene-DMR associations, expressions of the genes were detected in both tissues but significant associations were detected only in one tissue. This suggests the full set of genes with expression levels influenced by DNA methylation variation can only be documented through the analysis of many different tissues or growth conditions.

We assessed whether the DMRs that were significantly associated with gene expression were enriched for associations with SNPs. A subset (42–71%) of the DMRs that are associated with gene expression also have significant association with SNPs, with higher proportions for CG and CHG DMRs than for CHH DMRs (Fig. 5f). The remaining 29–58% of the DMRs associated with gene expression are not significantly associated with SNPs. Similar patterns were found for context-specific DMRs (Additional file 1: Figure S6e). Together, these results suggest that DMRs, whether or not tagged by SNPs, can explain variation in gene expression.

### A causal relationship between DMRs and variation in gene expression

Differential DNA methylation could be a cause or a consequence of differential gene expression. In an attempt to address this question, we compared two models using the Mendelian randomization test (Additional file 1: Figure S7a). The first model assumes a DMR is a cause of differential gene expression. We selected instrumental SNPs that show strong association with DMRs but not with gene expression, so that the effect of the SNP on gene expression was because of the combined effect of SNP on DMR and the effect of DMR on gene, which is called the predicted effect. We then compared this predicted effect with the observed effect. Interestingly, there is a strong correlation between the predicted effect and the observed effect. In contrast, the correlation is much smaller in a second model where DMR is a consequence of differential gene expression. The difference in correlation between the two models is observed in both the kernel and leaf tissues (Additional file 1: Figures S7 and S8). It is also observed for each of the three sequence contexts irrespective of the direction of association and the distance between DMR and gene (Additional file 1: Figures S7 and S8). These support an idea that DMR is more likely to be a cause of differential gene expression.

Next, we set to validate the causative role of DNA methylation on gene expression by using methylome mutants. We took advantage of the RNA-seq data from kernel tissue of the *ddm1* double mutant which disturbs DNA methylome dramatically [37]. We assessed whether the genes detected as exhibiting natural variation for DNA methylation linked to DMRs in our population would also show changes in expression in the *ddm1* mutant. As the *ddm1* mutant has limited changes in CG and there is no available mutant in maize that disturbs CG methylation, we focused on CHG and CHH methylation. For CHG DMRs, 67% of DMR-gene associations could be supported in *ddm1* mutant with the direction of association being the same in the natural population and in the *ddm1* mutant, compared to ~ 50% for random control which uses the top 5000 non-significant DMR-gene associations (Additional file 1: Figure S7b). For CHH DMRs, 62% were supported. Interestingly, both negative and positive associations from natural variation could be supported by the changes in expression in the *ddm1* mutant. These results provide further support that DNA methylation can have both a negative and a positive causal effect on gene expression.

### Natural DMRs explain phenotypic diversity

**Fig. 6:**
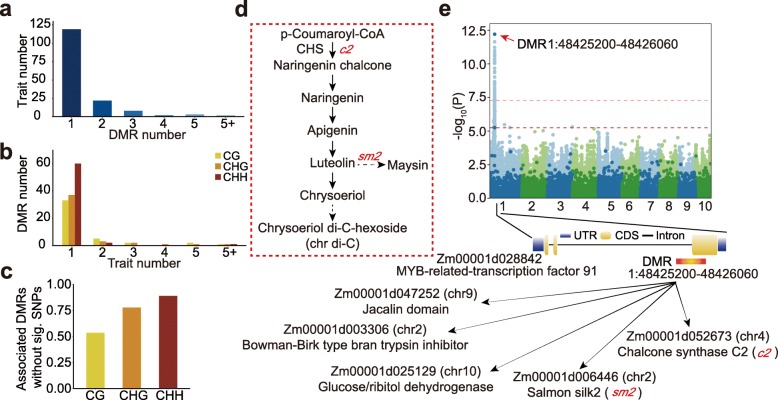
Association between DMRs and metabolic traits. **a** The distribution of the number of significant DMRs per trait. **b** The distribution of the number of associated metabolic traits per DMR. **c** The proportion of DMRs that are associated with metabolic traits but not with SNPs. **d** A simplified pathway for chrysoeriol di-C-hexoside (chr di-C) synthesis. The solid line represents a one-step reaction, and the dashed line represents a multi-step reaction. **e** GWAS result of chr di-C using either SNPs (light color) or DMRs (dark color). The horizontal dashed lines are the cutoff for SNPs (thinner) and DMRs (thicker)

If DMRs are tightly linked to nearby SNPs, then the potential phenotypic impact of this heritable information would be captured in SNP-based GWAS scans. However, if DMRs are not tightly linked to SNPs, then the variation in DNA methylation will reflect novel information not included in SNP-based scans. To identify whether DMRs can affect phenotypic variations both dependent upon and independent of SNPs, we performed GWAS using DMRs as independent variables and phenotypic data as dependent variables. We used the metabolic data that has been generated for this diverse panel in a previous study [38]. A total of 986 unique metabolic traits were measured in 3 independent environments. At a genome-wide significance level of 0.05/*N* (*N* is the number of DMRs which is 8864, 9759, and 5075 for CG, CHG, and CHH DMRs, respectively), 156 traits (15.8%) show significant associations with a DMR in at least 1 environment (Fig. 6a, Additional file 2: Table S4). The majority of the traits (76%) show associations with one DMR (Fig. 6a). There are 12 traits that are associated with the same DMR in at least 2 environments, suggesting that the effect of some DMRs is stable and reproducible. In total, 43 CG, 45 CHG, and 63 CHH DMRs were found to be significantly associated with metabolic traits (Fig. 6b). Some DMRs can associate with more than 1 metabolic trait, leading to a total of 250 DMR-trait associations. Interestingly, many of the significantly associated DMRs are not significantly associated with SNPs, with higher proportions for CHH DMRs than for CG and CHG DMRs (Fig. 6c). There are many traits that only show associations with DMRs but not with SNPs (Additional file 2: Table S4), suggesting that DMRs may be more predictable for phenotypic variation than SNPs for some traits.

One example is shown in Fig. 6d, e to demonstrate the effect of a DMR on phenotype. Chrysoeriol di-C-hexoside (chr di-C) is one type of flavonoid where chrysoeriol is linked to two sugar groups by an O-glycosidic bond. The flavone chrysoeriol is a derivative of chalcone (Fig. 6d). A total of eight significant associations with DMRs were identified for chr di-C content (Fig. 6e, Additional file 2: Table S4). The most significant associations were on chromosome 1 around 48.4 Mb. All three sequence contexts were found to be significant in two environments (Additional file 1: Figure S9a, Additional file 2: Table S4). The DMRs were located within a gene annotated as a MYB transcription factor which can bind promoters of flavonoid synthesis-related genes (Fig. 6e, Additional file 1: Figure S9b). This DMR was also found to associate significantly with the expression of five genes. Three of these five genes were annotated to have a role in flavonoid metabolism, including one glucose/ribitol dehydrogenase, one chalcone synthase (CHS), and salmon silk 2 (sm2). CHS is the major enzyme responsible for chalcone (Fig. 6d), which is the precursor for chrysoeriol. The *sm2* gene is also involved in the biosynthesis pathway of chrysoeriol (Fig. 6d). These five genes are located on different chromosomes from the DMR. It is possible that the transcription factor where the DMR is located can regulate the expression of these genes, leading to variations in chr di-C. This is supported by the fact that the expression of the five genes was associated with chr di-C content (Additional file 1: Figure S9c). The effect of this DMR may be related to genetic variation as many SNPs that are significantly associated with this DMR have been identified (Additional file 1: Figure S9d). These SNPs were located ~ 100 kb downstream from the DMR, and many of the SNPs were also significantly associated with chr di-C (Additional file 1: Figure S9b). However, the association for the DMR is the highest in one of the environments (Fig. 6e). These results suggest an important role of DNA methylation in contributing to phenotypic diversity.

## Discussion

### The genetic architecture of DMRs

In this study, we profiled DNA methylation across a diverse panel of 263 maize inbred lines. Extensive variation was found in all three sequence contexts across the ten maize chromosomes. One interesting discovery is that there is no obvious natural mutant with compromised DNA methylation in this panel that includes abundant genetic diversity. All 263 diverse inbreds have similar levels of DNA methylation, showing either high or low methylation at specific loci. The lack of distal hotspots that are associated with methylation machinery provides further support of the lack of natural variation for DNA methylation machinery. This is in contrast to studies in *Arabidopsis*. For example, the *VIM* gene, which controls for CG methylation, was identified in a natural *Arabidopsis* accession that shows genome-wide loss of DNA methylation [33]. The *CMT2* gene, which controls CHH methylation in heterochromatic regions, also has loss-of-function genetic variations in natural accessions [17, 32]. The lack of natural mutant of DNA methylation in maize suggests an important role of DNA methylation in plants with large genomes. This is well supported from studies in rice and maize, which show that genome-wide loss of DNA methylation leads to severe phenotypic effects and seed lethality in many cases [3–6, 39, 40].

Similar to prior results in other plant species [12, 16–18], a subset of regions with variable methylation can be associated with SNPs. This is true for all sequence contexts with variability in the proportion of DMRs for different sequence contexts that can be linked to SNPs. However, a large proportion (> 60%) of DMRs are not tagged by SNPs, suggesting that there is unique information in the DNA methylome. This was further supported by the observation that DNA methylation that is not associated with SNPs can effectively differentiate maize subgroups (Additional file 1: Figure S3b). These results support the concept that DMR can carry unique heritable information independent of genetic variation and that this information would not be captured in current SNP-based GWAS approaches. We should note, however, lack of association with SNPs does not necessarily mean that these DMRs are purely epigenetic. Low frequency SNPs which by design are not considered in GWAS could be associated with DMRs. Similarly, structural variants, which show low LD with SNPs, could also be associated with DMRs (Additional file 1: Figure S5a). We observed that DMRs with high MEF are more likely to associate with genetic variations, but we do find DMRs with high MEF that do not associate with genetic variations even after we consider the structural variants (Additional file 1: Figure S5b). These non-tagged DMRs with a relatively high MEF should be good candidates for pure epigenetic variants.

It is worthwhile to note that the capture-based method to assay DNA methylation levels in this study will have some limitations in monitoring genome-wide rates or variability and detecting the full set of information contained in the methylome. The capture-based approach focuses on a limited number of regions throughout the maize genome. These regions were chosen based on our previous knowledge of DNA methylation in maize genome [13, 30]. We purposely chose many regions with variation in DNA methylation that can be uniquely mapped across a very diverse set of lines. These regions represent portions of the genome that are more conserved across different maize lines. Considering the limited portion of the genome represented by our capture probes (15 Mb, or 0.6% of the entire genome), it is very likely that there are more regions showing variation in DNA methylation, and pure epigenetic information with important biological significance may widely occur in maize.

### DNA methylation contributes to differential gene expression

Both positive and negative associations between DNA methylation and gene expression have been proposed [13, 23, 30, 41]. We found that this relationship depends heavily on sequence contexts and the position of DMRs relative to gene TSS. DMRs located within 1 kb of the TSS tend to be enriched for negative associations for CG and CHG methylation and positive associations for CHH methylation. Another interesting question is whether differential methylation is a cause or consequence of differential gene expression. Previous studies have shown that loss/gain of DNA methylation in methylome mutants is usually accompanied by changes in gene expression [16, 42–44]. This suggests that changes in DNA methylation can be a cause of differences in gene expression. A genome-wide assay of natural variation of DNA methylation on gene expression in model plant *Arabidopsis* also suggests that DNA methylation is more likely the cause rather than the consequence of variation in gene expression [23]. However, there are also examples where DNA methylation is a consequence of differential gene expression [45]. Our genome-wide analysis favors the possibility that some DMRs are causes of variation in gene expression. The negative effect of DNA methylation on gene expression is likely due to a repressive chromatin environment associated with DNA methylation, while the positive effect of DNA methylation is likely attribute to the recruitment by DNA methylation of specific transcription factors to enhance gene expression [46].

### Epigenetic information contributes to phenotypic diversity

Many agronomic traits have a complex genetic basis with numerous minor effect QTLs [47–49]. The fact that only a portion of the phenotypic variance of these traits can be explained by SNPs has led to the suggestion that other types of heritable information may be involved. The growing use of SNP information for performing genomic selection in plant breeding has increased our reliance upon SNPs to explain phenotypic variation. We were interested in exploring the potential for DNA methylation to provide information about gene expression or phenotypic differences that may not be present in SNP profiles. There are examples for which DNA methylation, rather than genetic variation, at specific loci is the cause of a phenotypic change [50]. It was reported that DNA methylation can lead to phenotypic diversity independently of genetic variation based on QTL mapping using epi-recombinant inbred lines that share same genetic background but show variations in DNA methylation patterns [51, 52]. Here, we found that many of the DMRs were not captured by SNPs, some of which also show significant associations with gene expression and phenotypic diversity. Our results suggest that DNA methylation can affect phenotypic diversity under at least two circumstances. First, DNA methylation is a bridge between genetic variation and phenotype. In this scenario, a genetic difference (SNP or structural variant) is the cause of phenotypic variations and methylation changes are trigged by the genetic variation. Second, pure epigenetic information is contained within DMRs that are independent of genetic variation. There are many metabolic traits for which significant associations were only identified for DMRs but not for SNPs. Our study highlights the potential for DNA methylation variation that is not identifiable through SNPs and for this variation to influence gene expression and traits.

## Conclusions

In conclusion, DNA methylation was investigated at a population level in maize. Abundant variation in DNA methylation was identified, only some of which is effectively captured by SNP associations. Variations in DNA methylation can separate maize subgroups, associate with differential gene expression, and contribute to phenotypic diversity. This study represents the first effort to perform genome-wide association analysis using epigenetic data (DNA methylation) in a crop species. The findings that DMRs not tagged by genetic variation are prevalent and can cause phenotypic variation suggest that DNA methylation is a candidate to explain a portion of the heritability that is not effectively captured by SNPs.

## Methods

### Materials

The maize inbred lines used in this study are from a global collection and represent a wide range of diversity [53]. These lines were grown using standard greenhouse conditions until V3, and the third leaf was collected and frozen in liquid nitrogen. Genomic DNA was extracted using the standard cetyl-trimethyl-ammonium bromide method. Two approaches were used to verify material authenticity. First, five polymorphic InDel markers were used to genotype the entire panel, and the results were compared with that from previous genotyping. Only lines with consistent genotyping were kept. Second, SNPs were de novo called based on the sequencing data generated in this study using BS-SNPer [54] and were compared with the SNP data from a previous study [55]. The genotypes with consistency of more than 90% were kept, resulting in a total of 263 lines.

### Library preparation, sequencing, and mapping

The design of probes for the capture regions can be found in a previous study [29]. Briefly, 15.7 Mb of the maize genome was used for probe design based on B73 reference genome (AGPv2). These regions include all the regions from our first version of capture probes [28], as well as additional regions that are selected based on our study of DNA methylation in maize. These additional regions mainly include DMRs between genotypes (B73 and Mo17 or Oh43), DMRs among five tissues (seedling leaf, mature leaf, shoot apical meristem, anther, and immature ear), DMRs identified during tissue culture, promoters of potential hidden genes which are defined as genes that have high DNA methylation in B73 genome and low/no expression in various B73 tissues, and mCHH island. Information of the capture regions can be found on Data Repository for University of Minnesota [56].

Bisulfite-converted sequencing libraries were constructed using a previously published method for capturing specific regions [28]. The libraries were sequenced on HiSeq 2500 platform using 125 cycles and paired-end mode. After trimming adapters by Trim Galore [57], reads were mapped to the B73 reference genome version 4 [31] using BSMAP allowing up to five total mismatches [58]. Reads that are mapped uniquely were kept. Duplicate reads and reads that are not properly paired were also removed using Picard Tools [59] and BamTools [60]. The methylation levels at each individual cytosine were called using methratio.py in BSMAP.

### DMR identification

The DMRs among all the genotypes were identified using a two-step method. In step 1, DMRs were identified between two lines. The maize genome was divided into non-overlapping 20 bp windows, and the methylation levels of each 20 bp window that have cytosine sites with at least 2× coverage were calculated for each line. Sixteen lines with high read coverage and genetic diversity were selected as group I, with all the other lines as group II. Each line from the first group was then compared with each of the line from the second group. For each pair-wise comparison, the 20-bp windows with CG and CHG difference of greater than 60% were kept. For CHH, the windows that meet the following criteria were kept: over than 20% difference between the contrasted genotype, having one genotype of less than 5% methylation, and the other genotype of greater than 25% methylation. The 20-bp windows that are within 50 bp of each other and have the same direction were then merged for each comparison, and the merged regions with at least three continuous 20-bp windows with data were classified as DMRs.

In step 2, the DMRs from each pair-wise comparison were merged using the following pipelines. First, DMRs within 200 bp of each other were merged using BEDTools [61]. Second, the merged DMRs were divided into 20 bp windows and the percentage of contrasting pairs with significant difference in DNA methylation for each 20 bp window was calculated. This percentage was then normalized to the window with the highest percentage. The window with the normalized value of less than 0.4 was dropped, and the DMRs with at least three continuously eligible windows were kept. Third, each remaining DMR was required to meet the following criteria in > 60% of the lines: ≥ 2× coverage, ≥ 6 cytosine sites, and ≥ 2/3 of the cytosines within the region were covered. The above procedure was done separately for the three sequence contexts. Lastly, regions were classified as CG or CHG DMRs if the difference in methylation was greater than 60% between the second top highest and lowest lines for CG or CHG contexts, and was called as CHH DMR if the second highest line having a CHH level of > 25% and the second lowest line having a CHH level of < 5%. This would eliminate DMRs that are unique to a single line.

To define context-specific DMRs, the following criteria were used. For CG DMRs, if CHG methylation difference was greater than 60% and the squared Pearson correlation coefficient (*R*^2^) between CG and CHG levels were more than 0.8, the CG DMR was called as CG_CHG DMRs. If CHG methylation difference was less than 20% and *R*^2^ was less than 0.2, the CG DMR was called as CG_only DMR. Similarly, for CHG DMR, if CG methylation difference was greater than 60% and the *R*^2^ between CG and CHG levels were more than 0.8, the CHG DMR was called as CG_CHG DMRs. If CG methylation difference was less than 20% and *R*^2^ was less than 0.2, the CHG DMR was called as CHG_only DMR.

### Comparison of relatedness using SNPs or DNA methylation

Two methods were used to compare the individual relatedness generated by SNP and DNA methylation levels, respectively. First, the R package, pcaMethods, was used to perform PCA analysis [62]. The algorithm was ppca, and the number of components was set as 3. Second, GCTA was used to calculate individual relatedness based on SNPs and OSCA was used to generate individual relatedness based on DNA methylation data [63, 64]. The *R*^2^ of the two matrices with the diagonal value being removed was used to quantify the relationship between SNP and DNA methylation. The data for the three DNA methylation sequence contexts were calculated either separately or together.

### Identification of subgroup-specific DMRs

A one-way ANOVA was conducted to compare DNA methylation levels among three maize subgroups: SS, NSS, and TST. The significant DMRs at the *P* < 0.001 level were selected for post hoc comparisons using the Tukey HSD test. The DMRs that are significant at the *P* < 0.001 level among any two subgroups were defined as subgroup-specific DMRs.

### MEF calculation

For CG and CHG DMRs, if the difference in methylation was greater than 60% between any two lines, the line with higher methylation was assigned into “High” group and the other line was assigned into “Low” group. DMRs for which > 50% of the lines could be assigned to either “High” or “Low” group were kept. The MEF was calculated by the number of lines in the group with fewer lines divided by the sum of lines in the two groups. For CHH DMRs, the methylation difference was that the higher line having a CHH level of > 25% and the lower line having a CHH level of < 5%. The rest is similar as CG and CHG DMRs.

### Methylation QTL mapping

An integrated map of 1.25 million SNPs from a previous study [55] was converted to B73 version 4 coordinates using alignment of the sequences around the SNPs. The SNPs were retained if the position of the B73 V4 reference genome has exactly the same nucleotide as the B73 SNP from the conversion based on alignment. The SNPs were further filtered to retain those that have a minor allele frequency (MAF) of greater than 0.05 in the lines with methylation data. For each DMR, the methylation value was normalized using rank-based inverse normal transformation. A mixed linear model was used to perform GWAS by controlling for population structure and family relatedness [65]. The kinship was calculated by EMMAX, and the population structure was estimated by ADMIXTURE [66]. A Bonferroni-corrected *P* < 5.15 × 10^−8^ (0.05/*N*, *N* = 971,267) was used to determine significant associations. The association between structural variants and DMRs was calculated by the same way with a Bonferroni-corrected *P* < 1.84 × 10^−5^ (0.05/*N*, *N* = 2711) for structural variants identified based on comparison between B73 and SK and *P* < 2.01 × 10^−5^ for structural variants identified based on comparison between Mo17 and SK (0.05/*N*, *N* = 2484). As some associations may be caused by SNPs in LD, two criteria were used to filter these associations. First, LD analysis was performed using Haploview for all significant SNPs and only independent SNPs (*r*^2^ < 0.1) were kept [67]. The remaining SNPs should not be in LD with any other SNPs that are associated with the same DMR. Second, the median values of methylation for each haplotype was calculated and only associations having methylation difference greater than 0.05 were remained. According to the distance between DMRs and the associated SNPs, SNPs within 10 Mb of DMRs were defined as local SNPs and SNPs that are on another chromosome were distal SNPs. The SNPs that are on the same chromosome of DMRs but > 10 Mb away were defined as unclassified SNPs. DMRs that only had local SNPs were defined as “Local_only” type. DMRs with only distal associations were defined as “Distal_only.” DMRs that have both local and distal SNPs were defined as “Both.” DMRs that have unclassified SNPs were defined as “Unclassified.” DMRs that do not have associated SNPs were defined as “None.”

### Chromatin features of context-specific DMRs

The ChIP-seq data of histone modifications, H3K9me2 (SRR1482372) [68] and H3K27me3 (SRR5436222) [69], were downloaded from NCBI. The sequencing reads were processed using Trim Galore to remove adapters and nucleotides with bad quality. Reads that passed quality control were mapped to the reference genome of B73 version 4 using Bowtie2 [70] with default parameters. The metaplots showing read coverage around context-specific DMRs were generated using deepTools [71].

### Association between DMRs and quantitative traits including gene expression and metabolic traits

A single-marker test based on mixed linear model was used to analyze the association between DMR and gene expression/metabolic traits. Metabolic traits were from the study of Wen et al. [38]. There are 192 lines that have both metabolic data and methylation data. Two RNA-seq datasets were utilized, one with RNA-seq data for kernels 15 days after pollination [72] and another with RNA-seq data for leaf tissue. For kernel tissue, there are 193 lines that have both expression and methylation data. For leaf tissue, there are 108 lines with expression and methylation data. The RNA-seq reads were re-mapped to B73 v4 genome using TopHat2 [73] with the default parameters. Only the genes that have expression and methylation data in at least 60% of the lines were used. A two-step residual model was applied. In the first step, gene expression or metabolic traits (*Y*) were normalized using rank-based inverse normal transformation method. The normalized value was then fit in the following model to get a residual expression level (*Y*′) after controlling for the effect of population structure (*Q*) and individual relatedness (*K*).

$$ Y=Q+K+\upvarepsilon 1 $$

In step 2, the newly calculated *Y*′ was then used as the dependent variable to fit a linear regression model with DNA methylation (*X*) as the independent variable.

$$ Y^{\prime }=\beta X+\upvarepsilon 2 $$

The resulting *P* value was corrected using the Bonferroni method (*P* < 0.01/*n*), and the value was 1.13 × 10^−6^ for CG, 1.02 × 10^−6^ for CHG, and 1.97 × 10^−6^ for CHH. The direction of association was inferred from *β* value.

### Mendelian randomization

In order to investigate whether DNA methylation is a cause or a consequence of differential gene expression, Mendelian randomization (MR) test was performed using the significant DMR-gene expression associations. Only the associations where the DMR and the gene are within 1 Mb of each other were used. In the model where DMR is a cause, we identify instrumental SNPs that are significantly associated with DNA methylation, but not associated with gene expression. All the significant SNPs within 1 Mb of DMRs were extracted, and the associations between these SNPs with expression were calculated using the mixed model adjusting for *Q* and *K*. The SNPs that are significantly associated with gene expression (*P* < 0.05/DMR number) were filtered out, and the one with the most significant association with DNA methylation from the remaining SNPs was kept. The observed effect of the SNP on expression from the mixed model was then compared with the predicted effect which was calculated using the following formula,

$$ {\beta}_{\mathrm{pred}}={\beta}_{\mathrm{expr}\sim \mathrm{DMR}}\times {\beta}_{\mathrm{DMR}\sim \mathrm{SNP}} $$

In the consequential analysis, instrumental SNPs were required to be associated with gene expression but not with DMR. First, all significant SNPs within 1 Mb of genes were extracted. Then, the SNPs with significant associations with DMR (*P* < 0.05/gene number) were filtered out, and then, the most significant SNP with gene expression was chosen from the remaining SNP list. We then calculated the effect of gene expression on DMR using gene expression as independent variable and DNA methylation as response. The observed effect of SNP on DMR was from the model DMR~SNP. And the predicted effect was calculated as follows:

$$ {\beta}_{\mathrm{pred}}={\beta}_{\mathrm{expr}\sim \mathrm{SNP}}\times {\beta}_{\mathrm{DMR}\sim \mathrm{expr}} $$

The above analysis was performed separately for each of the three sequence contexts.

## Supplementary information

**Additional file 1: Figure S1.** DMR validation. Comparison between DNA methylation of the capture based assay and the whole genome bisulfite sequencing assay (WGBS) in DMRs between B73 and Mo17. **Figure S2.** Distribution of DMR size and genomic features. **Figure S3.** DNA methylation can reflect genetic distances. **Figure S4.** DNA methylation levels in DMRs are different among maize subgroups. **Figure S5.** Summary of DMRs associated with SNPs and structure variants (SVs). **Figure S6.** The associations between gene expression and DNA methylation. **Figure S7.** DMRs affect gene expression as a cause. **Figure S8.** Mendelian randomization analysis. **Figure S9.** The association between DMR and metabolic trait chr di-C.

**Additional file 2: Table S1.** Summary of data generated in this study. **Table S2.** DMRs with significant SNPs. **Table S3.** Associations between DMR and gene expression in kernel and leaf. **Table S4.** Associations between DMRs and metabolic traits.

**Additional file 3.** Review history.

## Data Availability

The datasets generated during the current study has been deposited into the National Center for Biotechnology Information under project number PRJNA549173 [74]. The ChIP-seq data for H3K9me2 (SRR1482372) and H3K27me3 (SRR5436222) were downloaded from NCBI [68, 69].
