# Supplementary material for: Population-level analysis reveals the widespread occurrence and phenotypic consequence of DNA methylation variation not tagged by genetic variation in maize
Source: Genome Biol. 2019 Nov 19;20:243. doi: 10.1186/s13059-019-1859-0 (PMC6862797; doi:10.1186/s13059-019-1859-0)
Supplement: Supplementary file 1 — Additional file 1: Figure S1. DMR validation. Comparison between DNA methylation of the capture based assay and the whole genome bisulfite sequencing assay (WGBS) in DMRs between B73 and Mo17. Figure S2. Distribution of DMR size and genomic features. Figure S3. DNA methylation can reflect genetic distances. Figure S4. DNA methylation levels in DMRs are different among maize subgroups. Figure S5. Summary of DMRs associated with SNPs and structure variants (SVs). Figure S6. The associations between gene expression and DNA methylation. Figure S7. DMRs affect gene expression as a cause. Figure S8. Mendelian randomization analysis. Figure S9. The association between DMR and metabolic trait chr di-C. [file 13059_2019_1859_MOESM1_ESM.pdf]

Population-level analysis reveals the widespread occurrence and phenotypic consequence of DNA methylation variation not tagged by genetic variation

Jing Xu[a,1], Guo Chen[a,b,1], Peter J. Hermanson[c], Qiang Xu[a], Changshuo Sun[a], Wenqing Chen[a], Qiuxin Kan[a], Minqi Li[a], Peter A. Crisp[c,d], Jianbing Yan[a], Lin Li[a], Nathan M. Springer[c,e,*], Qing Li[a,*]

[a] National Key Laboratory of Crop Genetic Improvement, Huazhong Agricultural University, Wuhan 430070, China

[b] Institute of Nuclear and Biological Technology, Xinjiang Academy of Agricultural Sciences, Urumqi 830091, China

[c] Department of Plant and Microbial Biology, University of Minnesota, St. Paul, MN 55108, USA

[d] @pete_crisp

[e] @SpringerNathan

[1] Contributed equally

[*] Co-corresponding author

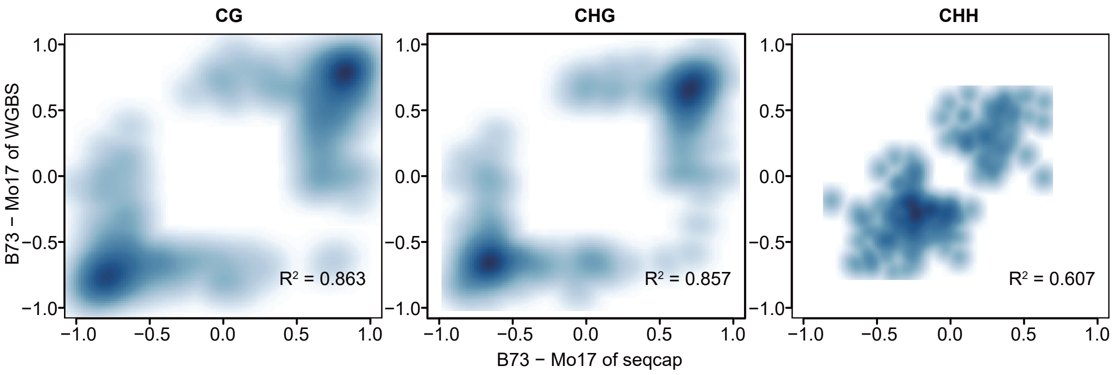

Figure S1. DMR validation. Comparison between DNA methylation of the capture based assay and the whole genome bisulfite sequencing assay (WGBS) in DMRs between B73 and Mo17. The x-axis is values of methylation levels in B73 minus Mo17 from the capture based assay and the y-axis is the corresponding from the WGBS assay.

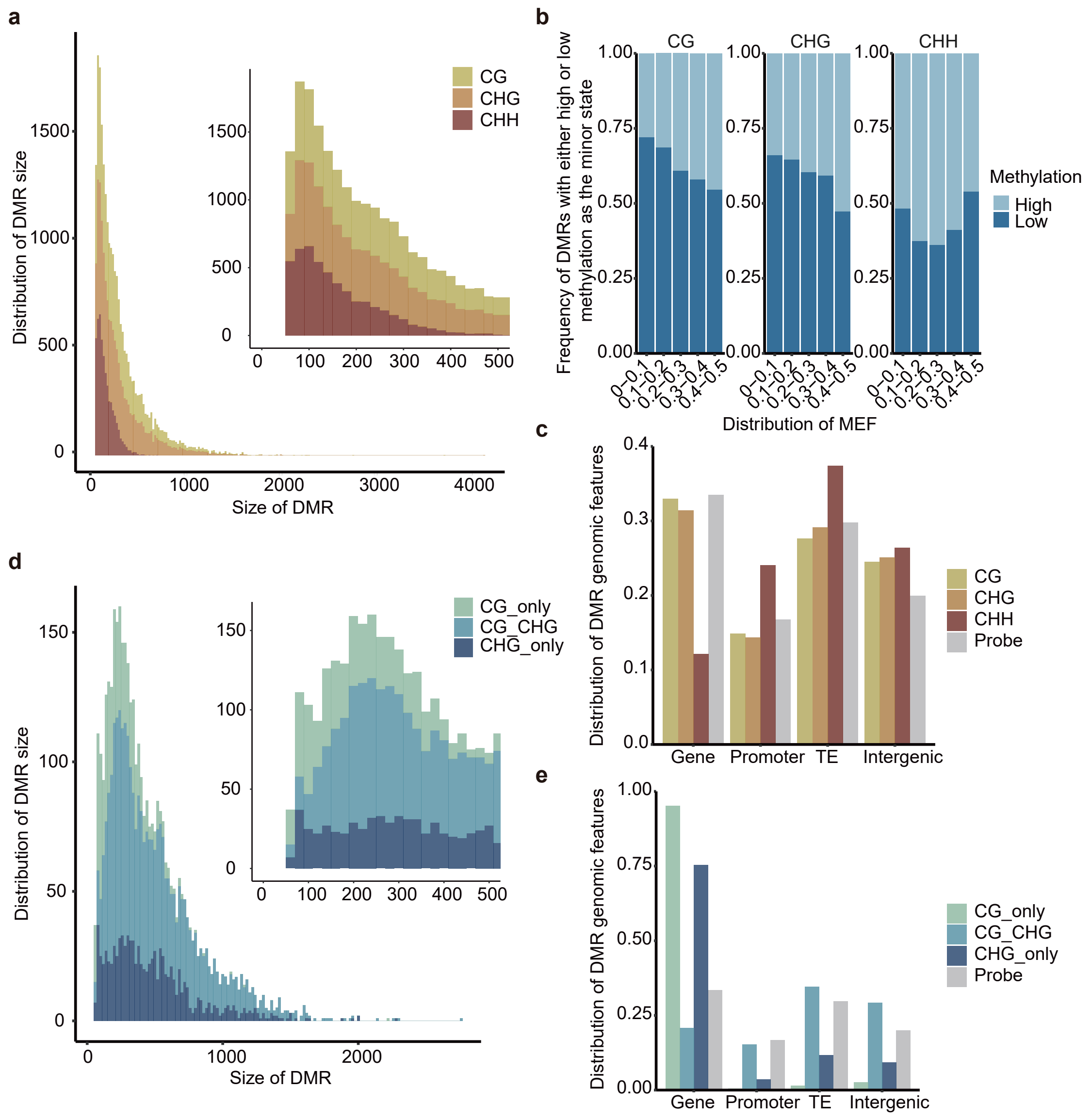

Figure S2. Distribution of DMR size and genomic features. **a** DMR size distribution for the three sequence contexts. **b** Frequency of DMRs with either high or low methylation as the minor state at different minor epiallele frequency (MEF) for the three sequence contexts. **c** The genomic feature that overlaps with DMRs for each of the three sequence contexts is shown. **d** Size distribution of context-specific DMRs. **e** The distribution of genomic annotations that overlap with context-specific DMRs is shown relative to the annotations for all probed regions (grey).

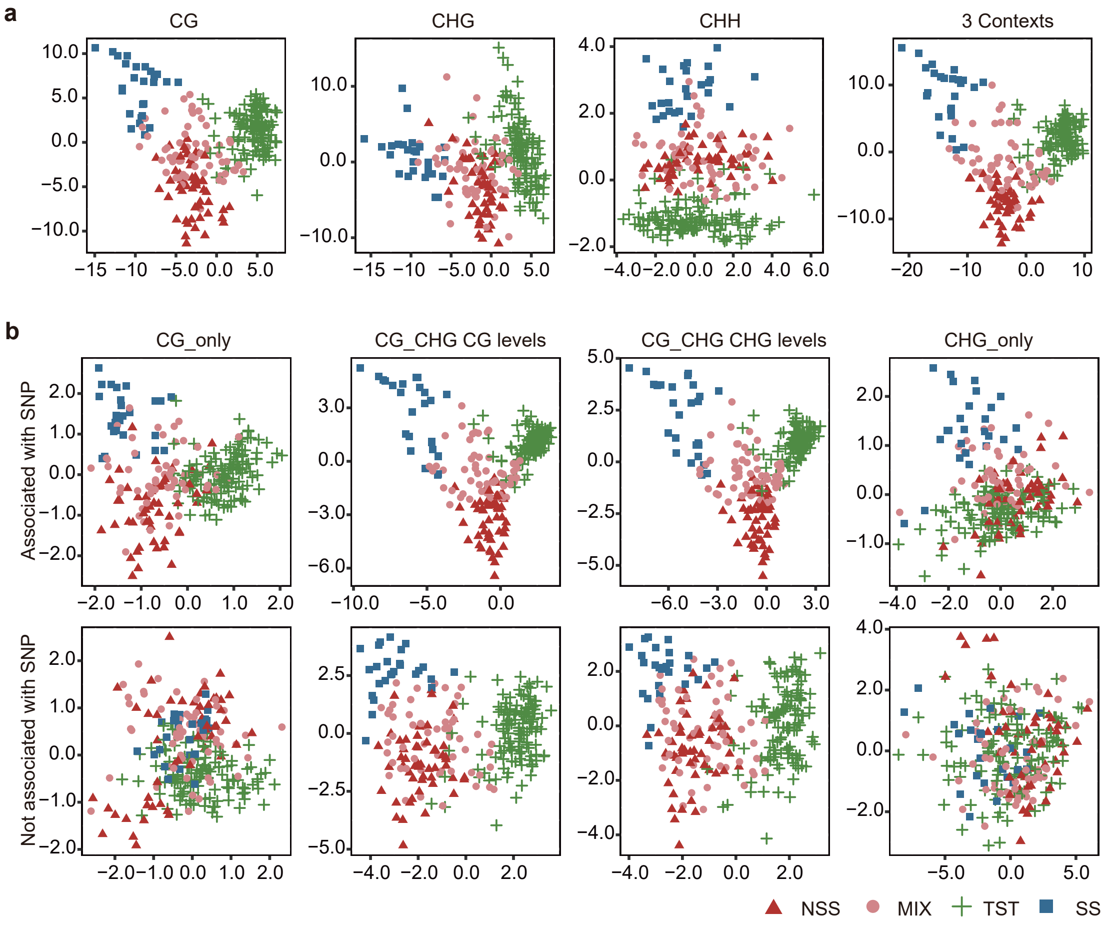

Figure S3. DNA methylation can reflect genetic distances. **a** PCA plots to differentiate maize subgroups using DNA methylation in the indicated sequence context. **b** PCA plots of context-specific DMRs with or without significant associations with SNPs. SS, stiff stalk; NSS, non-stiff stalk; TST, tropical or semi-tropical.

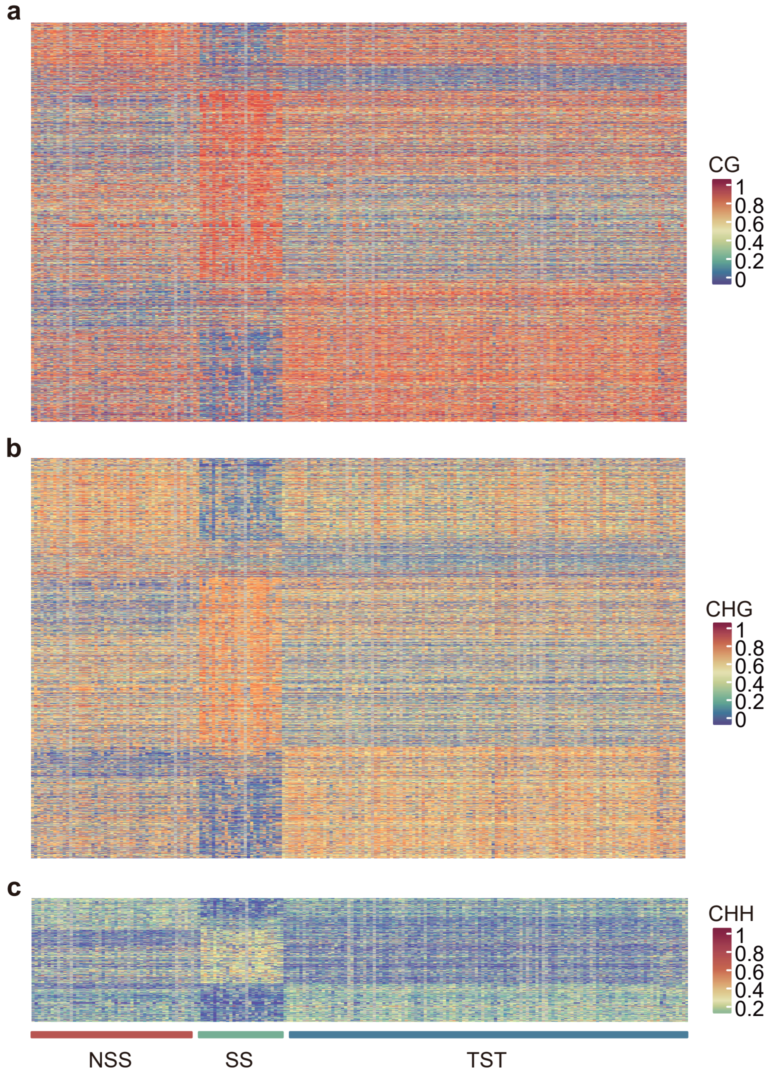

Figure S4. DNA methylation levels in DMRs are different among maize subgroups. **a** A total of 2129 CG DMRs are different among the three maize subgroups. **b** A total of 2154 CHG DMRs are different among the three maize subgroups. **c** A total of 659 CHH DMRs are different among the three maize subgroups. SS, stiff stalk; NSS, non-stiff stalk; TST, tropical or semi-tropical.

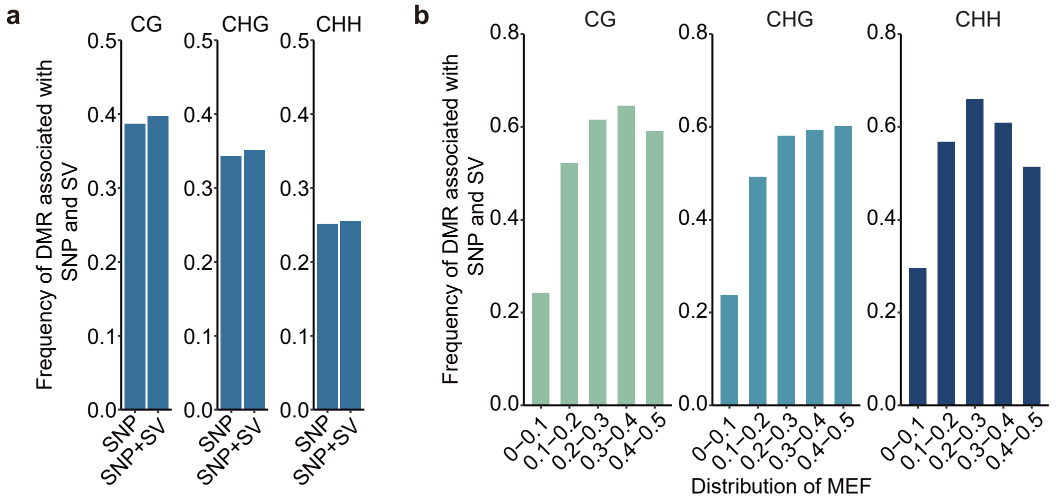

Figure S5. Summary of DMRs associated with SNPs and structure variants (SVs). **a** Frequency of DMRs associated with SNP/SV. **b** Frequency of DMRs associated with SNP/SV at different minor epiallele frequency (MEF).

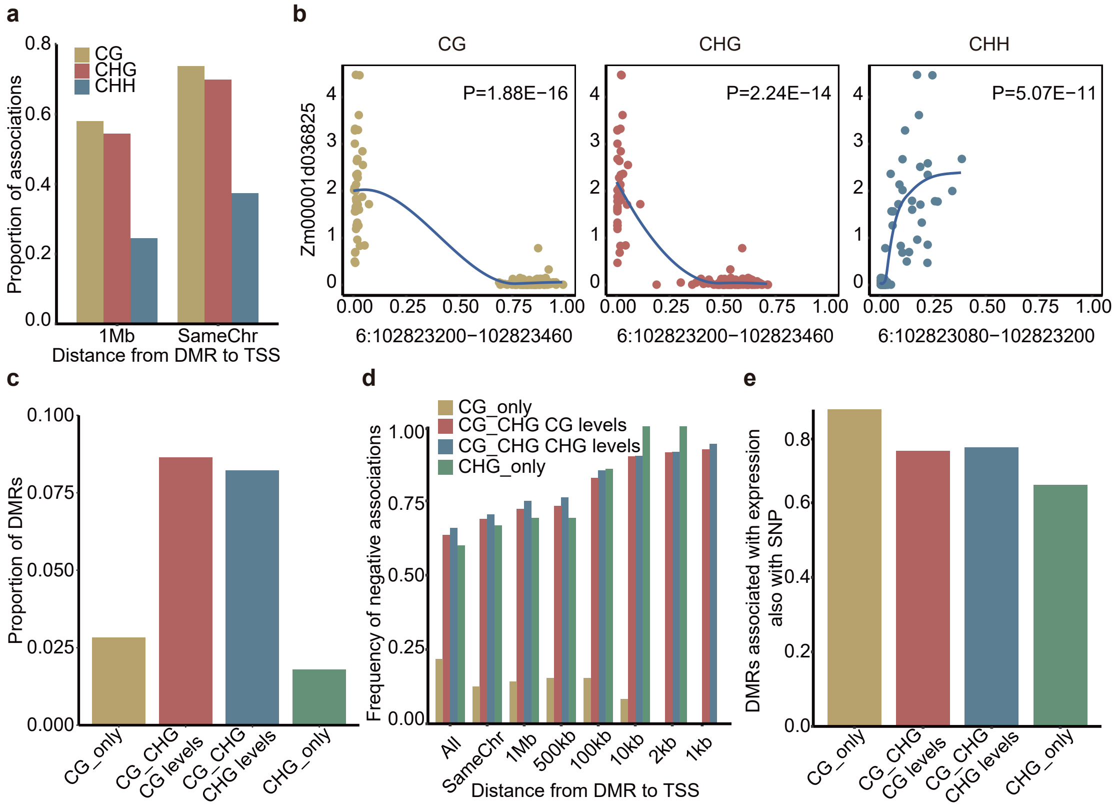

Figure S6. The associations between gene expression and DNA methylation. **a** The proportion of DMR-gene associations for which the DMR and the gene are located on the same chromosome (SameChr) or within 1 Mb of each other. **b** An example which shows that all three contexts within a DMR can associate with expression of the same gene. **c** The proportion of context-specific DMRs having associations with gene expression. **d** Summary plot to show the proportion of negative associations for context-specific DMRs with varying distance to gene TSS. **e** The proportion of context-specific DMRs that are associated with gene expression and with SNPs.

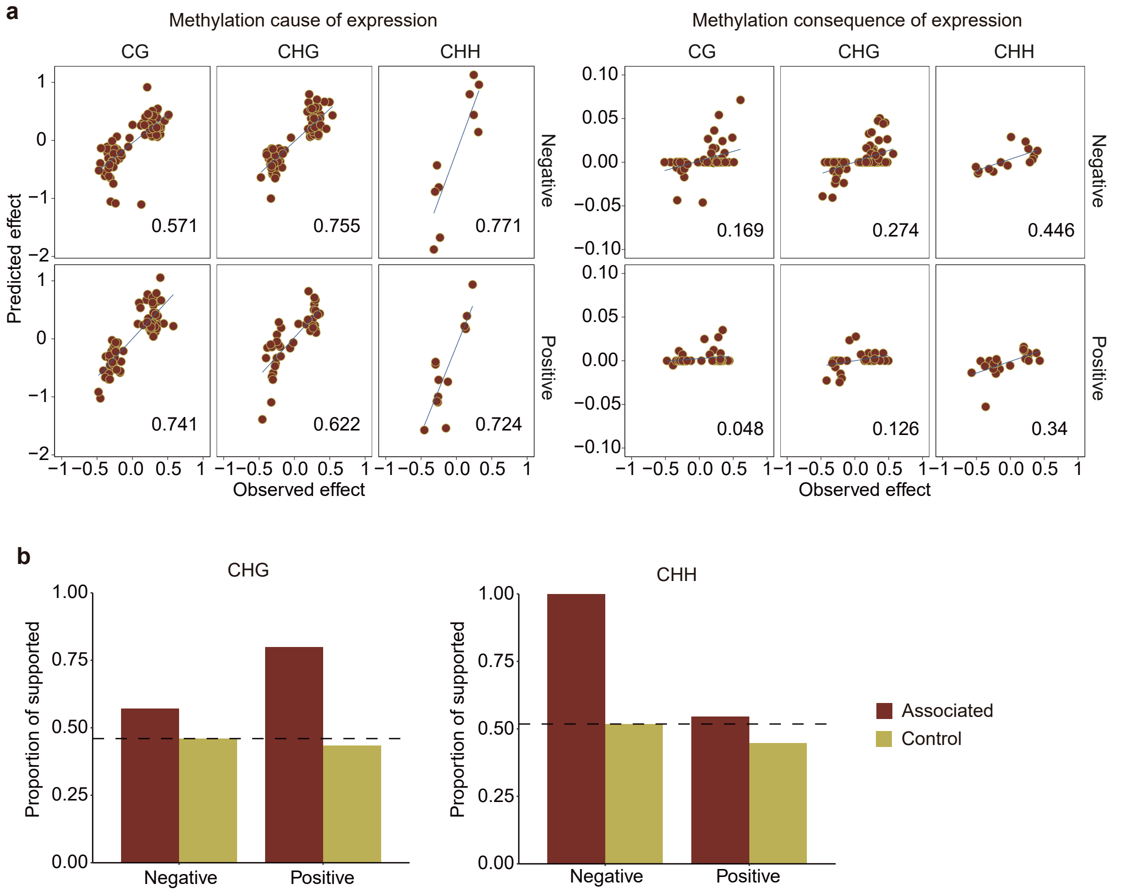

Figure S7. DMRs affect gene expression as a cause. **a** Results for the causality analysis of DNA methylation and gene expression. The squared Pearson correlation coefficient (R^2) between observed and predicted effect is shown in the right bottom of each plot. The blue line is the fitted curve of observed and predicted effect. **b** Validation of the causal effect of DMR on gene expression in *ddm1* mutant. Only DMR-gene pairs that show consistent direction of associations were considered “supported”. Control is the top 5,000 non-significant DMR-gene associations.

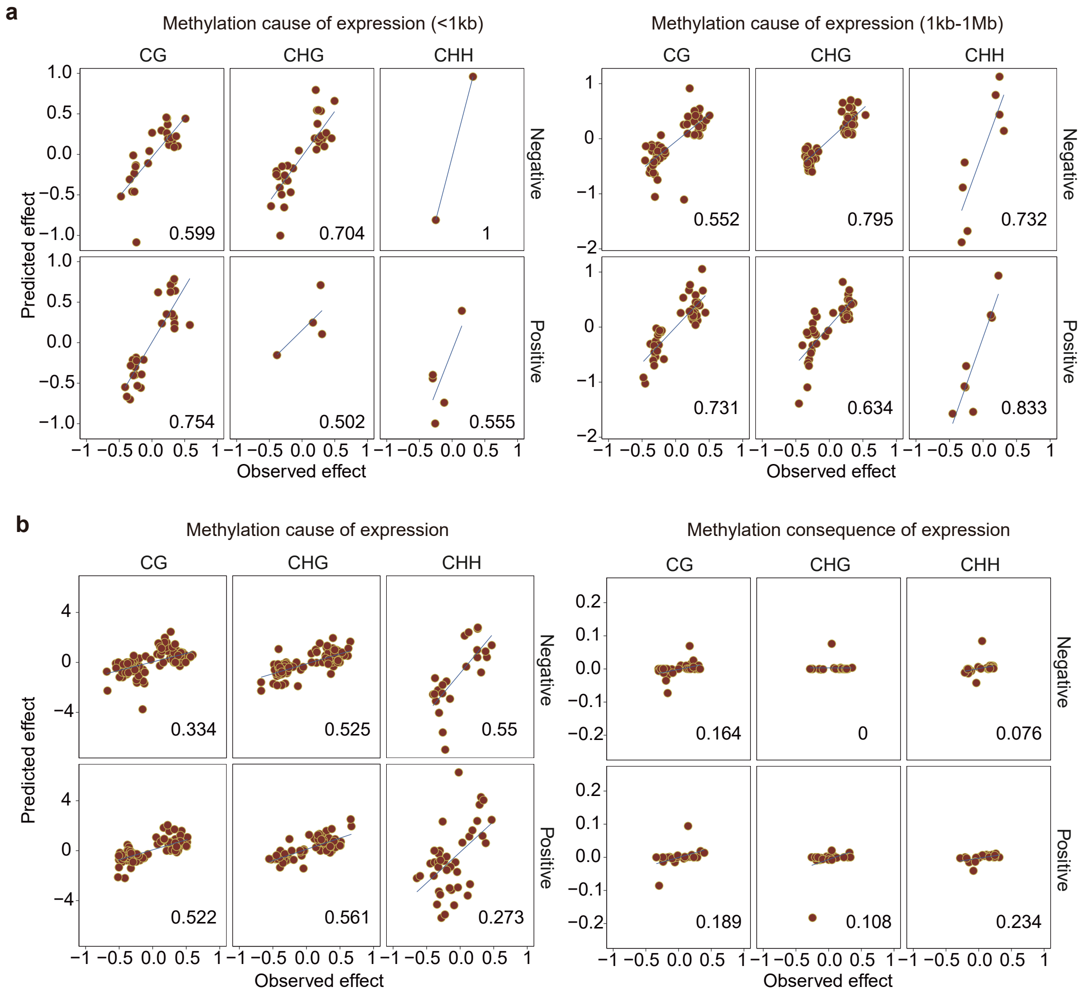

Figure S8. Mendelian randomization analysis. **a** Mendelian randomization test splitting by distance between DMR and gene in kernel. **b** Mendelian randomization test of all DMR-gene associations in leaf.

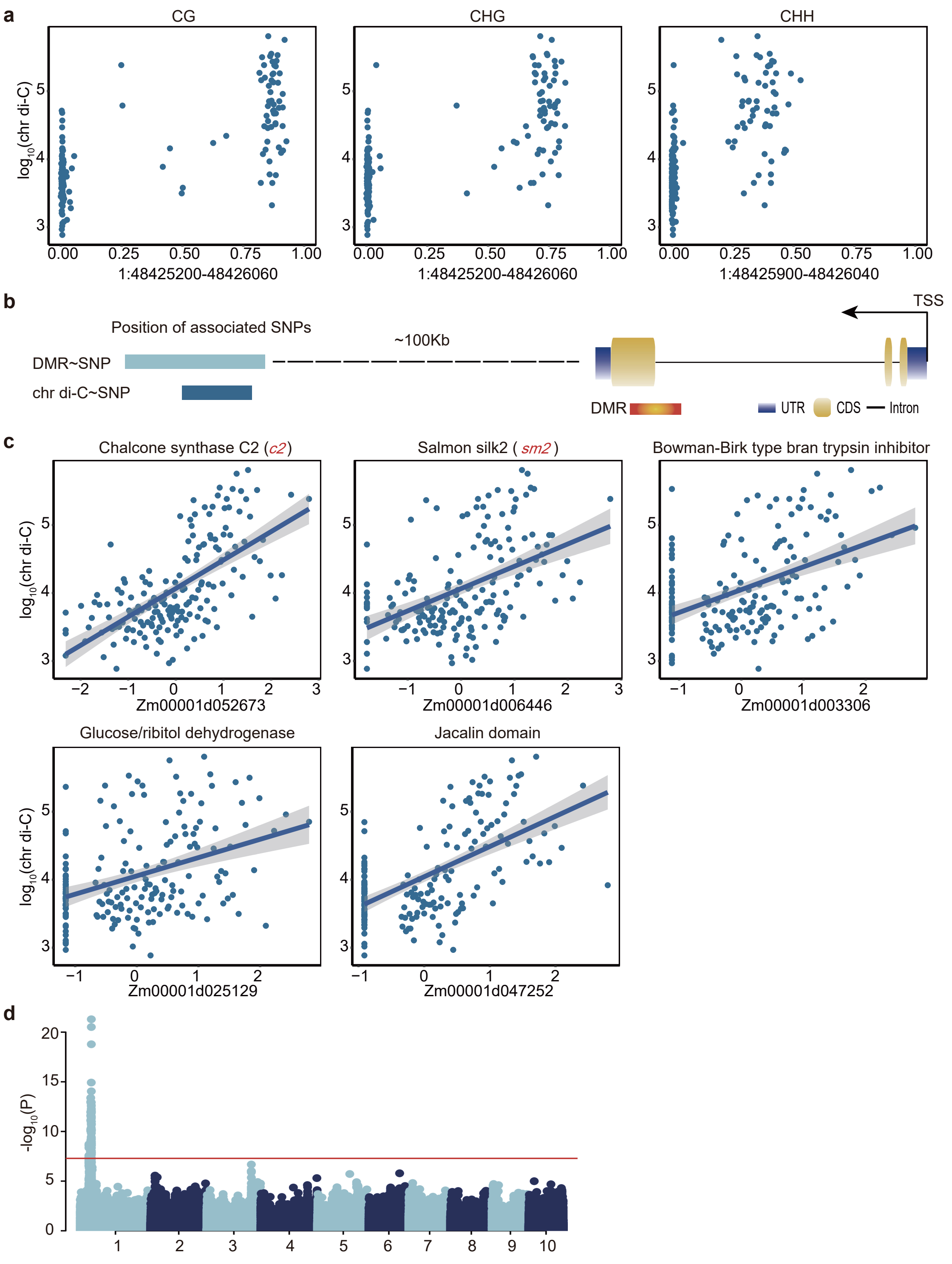

Figure S9. The association between DMR and metabolic trait chr di-C. **a** The correlations between chr di-C content and CG/CHG/CHH methylation of the DMR. **b** The relative position of the DMR, the SNPs associated with the DMR, and the SNPs associated with chr di-C content. **c** The associations between chr di-C content and the expression levels of the five genes that are significantly associated with the DMR. **d** The DMR affecting chr di-C is also associated with genetic variation.
