# Supplementary material for: Population-level analysis reveals the widespread occurrence and phenotypic consequence of DNA methylation variation not tagged by genetic variation in maize
Source: Genome Biol. 2019 Nov 19;20:243. doi: 10.1186/s13059-019-1859-0 (PMC6862797; doi:10.1186/s13059-019-1859-0)
Supplement: Supplementary file 3 — Additional file 3. Review history. [file 13059_2019_1859_MOESM3_ESM.docx]

Review History

**First round of review**

**Reviewer 1**

**Are you able to assess all statistics in the manuscript, including the appropriateness of statistical tests used?** No, I do not feel adequately qualified to assess the statistics.

**Comments to author:**

In this manuscript, Xu et al investigate in maize the extent and potential phenotypic contribution of DNA methylation variation that is purely epigenetic, i.e. that is heritable yet not determined by DNA sequence variation. To this end, they determined across 263 diverse maize inbred lines the methylation level at single cytosine resolution of >20 000 relatively well-conserved regions. These regions were isolated using capture-based method previously developed by the Springer lab.

The authors identified several thousands DMRs for each of the three sequence contexts (CG, CHG and CHH). CG and CHG DMRs do in fact mostly overlap and, as expected from previous work in maize as well as other plants, different types of DMRs are characterized by distinct features, such as low vs high abundance of matching small RNAs or differential targeting by the two DNA methyltransferases MET1 and CMT3. 

Nonetheless, using GWAS, the authors found no consistent association between individual DMRs and components of the DNA methylation machinery and indeed all maize lines show similar overall DNA levels and patterns. Moreover, the authors show that 60% of the DMRs do not have a significant association with any SNPs. Contrary to what the authors conclude though, this lack of association by no means proves that any of the relevant DMRs are purely epigenetic, i.e. not determined by sequence variation. Indeed, low frequency (<0.05 in the current study) SNPs are by design not considered in GWAS and could nonetheless be causal and so could structural variants. This last point is particularly important to consider, given the recent article by Yang et al (Nat. Genet 2019, 51:1052-1059), which provides evidence that in maize "21.9% of the common Structural Variants showed low linkage disequilibrium with nearby SNPs, suggesting they are a source of genetic diversity not discoverable by SNPs". 

The authors go on to show that the majority of DMRs for which a significant association with gene expression was detected are also significantly associated with SNPs. This observation, together with the considerations exposed above, considerably weaken the conclusion drawn by the authors that "DNA methylation may carry unique heritable information for explaining variation in gene expression that is not captured by SNPs". If anything, it favors instead the alternative view that most non-tagged DMRs are not bona fide epigenetic variants. In this context, determining whether or not DNA methylation is a cause or consequence of differential gene expression using DMRs that are either tagged or not tagged by SNPs makes little sense.

In a last part, the authors convincingly show that non-tagged DMRs can nicely complement GWAS, as a number of them were found to be significantly associatied with diverse metabolic traits. 

In summary, this manuscript presents a wealth of very interesting results that are grossly overinterpreted. Although the authors need to reconsider many of their conclusions, the quality of the work presented and of the results obtained should be of immediate interest to a large audience.

Specific points:
(1)     Part of the introduction appears poorly written compared to the rest of the manuscript and needs English editing. 

(2)     The introduction presents many concepts very naively.  The first sentence provides a good example of this. Also, there are still very few in vivo data supporting a direct role for DNA methylation in the masking or unmasking of cis-acting elements to specific transcription factors. Conversely, that "the effect of genetic variation on gene expression is only manifested at certain tissues or under certain environmental conditions" should shock no one and it is not clear why this should be mentioned in the present study. 

(3)     Missing heritability is still an issue eleven years after the first GWAS were performed and epiallelic variation as a potential (partial) cause has been amply discussed, but the current study unfortunately adds little novel information to this debate. A mention in the Discussion would suffice. 

(4)     Ref. 33 and 34 may not be the best choices for illustrating the existence of epiallelic variation. A general review, such as Quadrana & Colot, Ann Rev Genet 2016, could be better suited.  

(5)     The end of the introduction should be toned down: "We found evidence for many examples of pure epigenetic information stored in DNA methylation. We showed that DNA methylation can affect expression of many maize genes…. Furthermore, we showed that DNA methylation can affect phenotypic variation in maize…". 

(6)     It has been shown in Arabidopsis that CHH and CG DMRs typically represent DNA methylation losses or gains, respectively. Is this also the case in maize?

(7)     By design, most DMRs tagged by SNPs should have a relatively high Minor Epiallele Frequency (MEF). Thus, if they exist, non-tagged DMRs with a relatively high MEF should be good candidates for pure epigenetic variants.

(8)     The first sentence of the discussion should be toned down: "Our genome-wide analysis supports a causative role for many of the DMR-gene expression associations that were detected.

(9)     The second sentence of the discussion is very naïve. There are now tens if not hundreds of examples in the literature of a positive effect of DNA methylation on gene expression!

(10)     "DNA methylation is an ideal candidate". Why ideal, when epistasis, SVs, rare alleles with large effects, etc. are all equally plausible explanations for the missing heritability problem?

(11)     The conclusion should be toned down.

**Reviewer 2**

**Are you able to assess all statistics in the manuscript, including the appropriateness of statistical tests used?** Yes, and I have assessed the statistics in my report.

**Comments to author:**

The paper by Xu et al is a very timely and useful addition to the toolkit available to the maize community. The authors used a 'capture based assay' to profile DNA methylation patterns for a panel of 263 maize lines from divergent backgrounds. They reach a number of interesting conclusions, which are well supported by the data. 

First, they identify a large number of DMRs (differentially methylated regions) within the panel roughly 9000 for CG, 10,000 for CHG, and 5000 for CHH, a lot of them located in genes, which is consistent with the fact that most of the probes are enriched for genes. promoters, TEs and intergeneric regions are also well represented, meaning that the assay essentially captures sufficient diversity for proper analyses.

Second, and unsurprisingly, the authors never identified any variant for the core methylation machinery within the panel. This was to be expected, since altered methylation in maize is well know to induce significant phenotypic responses. Note also that maize does not have a CMT2 homologue that I'm aware of.

Third, they show that that DMRs are a good proxy for differentiating maize subgroups. Interestingly, CG methylation shows the highest correlations, consistent with the prevalence of CG methylation in the genome. It is also noteworthy that CG-CHG DMRs are likely better estimators of genetic distance, reflecting possibly the fact that, as seen Fig 1b, many such loci co-vary substantially. 

Fourth, The authors performed GWA with a set of 1million SNPs. Interestingly, most DMRs associated with only one SNP, and most SNPs with only one DMR. Figure 3d suggest that most are in fact quite local (<1M), CHH having the least. This suggests that distal associations are relatively rare.

Fifth, an interesting analysis of the correlation between H3K9me2 and H3K27me3 suggests that low levels of CG_CHG DMRs anti-correlate with K9, while high levels of CH_CHG correlates positively with K9. The reverse observations are made for K27 (low positively correlates, high negatively), which all makes sense I guess. 

Sixth, a GWAS analysis suggests that, as a rule, CG and CHG DMRs negatively correlate with gene expression. While the trends are clear, they are not absolutely clear-cut, and this figure may require additional explanation, in particular, the conclusion that gene expression and DNA methylation is dependent upon sequence context is not so obvious, based on the existing data. Note also that Fig S6b generates a bit of confusion, as the clouds are clearly divergent on the graph. While the p values are solid, the trend line is overly optimistic. 

Seventh, the authors used a cleaver strategy to assess whether methylation would be casual relative to gene expression, or a consequence of gene expression. The data indeed suggests that methylation is causal rather that a consequence of gene expression. This is an important result, which they further confirmed using ddm1 mutants. Here again, both negative and positive causal correlations are found, consistent with the overall patterns. 

Finally, GWAS analysis for metabolic traits is provided. It shows that DMRs are a good predictor of phenotypic diversity. A significant number of DMRs indeed associate with metabolic traits, and many of them do not associate with SNPs. A illustrative exemple is provided for one specific pathway, and suggest that one DMR in particular has a very significant effect, suggesting that DNA methylation contributes significantly to phenotypic diversity. 

The paper is clearly of outstanding interest to a broad audience of biologists, and I would strongly support its publication. Overall, this a great paper. There are, however, a number of things that should be clarified before publication.

One aspect that is a bit annoying is the correlation between the GWBS sequencing (B73-Mo17) versus the capture assay (B73-Mo17). While the trends are relatively convincing (R2 of 0,76 for CG and 0,70 for CHG) the correlation for CHH is relatively weak (R2 of 0,36), and I wonder whether this might be the reason why the subsequent analyses with the CHH DMRs show trends conflicting with the other contexts. This applies to Fig5 (positively correlated), Figure S7a (R2= 0,34), S7b (negative =1, positive =0,5), S8a (only two points), S8b (R2=0,55 for negative correlation, R2=0,27 for positive correlations). 

Similarly, I'm not sure that the Fig S5 is really necessary. While the trends are interesting, I'm not sure they had much to the paper. Also, I would suggest that the authors refrain from using the Arabidopsis nomenclature for the maize mutants. It remains unclear whether cmt3 or cmt1 are the homologues of Zmet5 or Zmet2, and there are excellent data sets available from both lead authors for these mutants. 

Finally, I remain a bit confused with the GWAS data correlating gene expression with DMRs. I think this is likely one of the most interesting part of the paper (together with the causal aspect of DNA methylation), but the authors clearly need to better explain their rational for reaching such conclusion.

Also, minor points:

There are a significant number of typos in the text and the figure legends. Please, revise as needed.

**Authors Response**

**Point-by-point responses to the reviewers’ comments:**

Reviewer #1: In this manuscript, Xu et al investigate in maize the extent and potential phenotypic contribution of DNA methylation variation that is purely epigenetic, i.e. that is heritable yet not determined by DNA sequence variation. To this end, they determined across 263 diverse maize inbred lines the methylation level at single cytosine resolution of >20 000 relatively well-conserved regions. These regions were isolated using capture-based method previously developed by the Springer lab.

The authors identified several thousands DMRs for each of the three sequence contexts (CG, CHG and CHH). CG and CHG DMRs do in fact mostly overlap and, as expected from previous work in maize as well as other plants, different types of DMRs are characterized by distinct features, such as low vs high abundance of matching small RNAs or differential targeting by the two DNA methyltransferases MET1 and CMT3.

Nonetheless, using GWAS, the authors found no consistent association between individual DMRs and components of the DNA methylation machinery and indeed all maize lines show similar overall DNA levels and patterns. Moreover, the authors show that 60% of the DMRs do not have a significant association with any SNPs. Contrary to what the authors conclude though, this lack of association by no means proves that any of the relevant DMRs are purely epigenetic, i.e. not determined by sequence variation. Indeed, low frequency (<0.05 in the current study) SNPs are by design not considered in GWAS and could nonetheless be causal and so could structural variants. This last point is particularly important to consider, given the recent article by Yang et al (Nat. Genet 2019, 51:1052-1059), which provides evidence that in maize "21.9% of the common Structural Variants showed low linkage disequilibrium with nearby SNPs, suggesting they are a source of genetic diversity not discoverable by SNPs".

*Response: This is a good point. We agree with the reviewer that “lack of association with SNP doesn’t imply pure epigenetic”. Many genetic variants such as structural variants (or TE insertions) may not be ‘tagged’ by SNPs. We addressed this comment in two ways in the manuscript. First, we conducted GWAS with the structural variants (SVs) data from the article by Yang et al (Nat. Genet 2019, 51:1052-1059). Indeed, there is an additional 0.3%-1% DMRs that don’t associate with SNPs but associate with SVs. We added these results in the revised manuscript. Second, we altered the language in this section to acknowledge that the failure to identify SNPs associated with the DMRs does not necessarily mean that it is purely epigenetic.*

The authors go on to show that the majority of DMRs for which a significant association with gene expression was detected are also significantly associated with SNPs. This observation, together with the considerations exposed above, considerably weaken the conclusion drawn by the authors that "DNA methylation may carry unique heritable information for explaining variation in gene expression that is not captured by SNPs". If anything, it favors instead the alternative view that most non-tagged DMRs are not bona fide epigenetic variants. In this context, determining whether or not DNA methylation is a cause or consequence of differential gene expression using DMRs that are either tagged or not tagged by SNPs makes little sense.

*Response: We would respectfully disagree with the reviewer here. Our conclusion that “DNA methylation may carry unique heritable information for explaining variation in gene expression that is not captured by SNPs” was not specifically stating that this information is epigenetic. As the reviewer points out in the first major issue that “nonSNP tagged” DMRs may indeed be due to genetic variation that is not captured by SNPs. However, most GWAS studies of traits (or molecular properties such as gene expression) rely upon SNPs. We were simply suggesting that DMRs have additional information that SNP surveys may not capture. In this specific instance we find that many of the DMRs associated with expression also are associated with SNPs (71% for CG, 66% for CHG and 42% for CHH) (Figure 5f). However, this leaves the remaining 29% (CG), 34% (CHG) and 58% (CHH) of DMRs that are associated with gene expression that are not tagged by a SNP. We may be mis-understanding the reviewers’ point here but it seems that this analysis does suggest that a substantial number of cases in which DMRs are associated with gene expression are cases in which the DMR is not effectively captured by SNPs.*

In a last part, the authors convincingly show that non-tagged DMRs can nicely complement GWAS, as a number of them were found to be significantly associated with diverse metabolic traits. In summary, this manuscript presents a wealth of very interesting results that are grossly overinterpreted. Although the authors need to reconsider many of their conclusions, the quality of the work presented and of the results obtained should be of immediate interest to a large audience.

*Response: Thanks for the positive comments about the overall results. We agree with several of the reviewers’ critiques of our interpretations and have toned down the interpretation of the results.*

Specific points: (1)Part of the introduction appears poorly written compared to the rest of the manuscript and needs English editing.

*Response: We have substantially modified the introduction, hopefully it is satisfactory now.*

(2)The introduction presents many concepts very naively. The first sentence provides a good example of this. Also, there are still very few in vivo data supporting a direct role for DNA methylation in the masking or unmasking of cis-acting elements to specific transcription factors. Conversely, that "the effect of genetic variation on gene expression is only manifested at certain tissues or under certain environmental conditions" should shock no one and it is not clear why this should be mentioned in the present study.

*Response: Thanks for the suggestions. We have modified the introduction and altered the sentences the reviewer noted. We apologize for the confusing of the sentence “the effect of genetic variation on gene expression is only manifested at certain tissues or under certain environmental conditions”; we want to show that more tissues may be needed to assay the association between DNA methylation and gene expression. We modified the sentence to make it clearer. Now it reads “However, these studies assayed gene expression in one tissue. It is possible that more genes for which expression are associated with DNA methylation can be identified if more tissues were assayed, as has been found for genetic variation”.*

(3)Missing heritability is still an issue eleven years after the first GWAS were performed and epiallelic variation as a potential (partial) cause has been amply discussed, but the current study unfortunately adds little novel information to this debate. A mention in the Discussion would suffice.

*Response: This is a fair suggestion. We deleted the paragraph on “missing heritability” from the introduction and moved part of its contents into Discussion.*

(4)Ref. 33 and 34 may not be the best choices for illustrating the existence of epiallelic variation. A general review, such as Quadrana & Colot, Ann Rev Genet 2016, could be better suited.

*Response: Thanks for the suggestion. We added citation of the suggested review paper.*

(5)The end of the introduction should be toned down: "We found evidence for many examples of pure epigenetic information stored in DNA methylation. We showed that DNA methylation can affect expression of many maize genes…. Furthermore, we showed that DNA methylation can affect phenotypic variation in maize…".

*Response: We modified these sentences and now they read “We found evidence for many examples of differentially methylated regions (DMRs) that contain information that is not fully captured using SNPs. We showed that variation in DNA methylation is associated with variation in gene expression, and this association is dependent upon sequence contexts as well as the position of DMR relative to gene transcriptional start site. Furthermore, we showed that variation in DNA methylation is associated with phenotypic variation in maize and can explain a portion of the heritability of some metabolic traits.”*

(6)It has been shown in Arabidopsis that CHH and CG DMRs typically represent DNA methylation losses or gains, respectively. Is this also the case in maize?

*Response: It is difficult to assess whether DMRs represent methylation losses or gains in maize because the ancestral state of DNA methylation is unclear. We looked at which methylation type tends to be the minor state for CG, CHG and CHH (Figure S2b). For CG and CHG DMRs, the majority of DMRs tend to have low methylation as the minor states. For example, for the CG DMRs with MEF between 0-0.1, ~75% of them have low methylation as the minor methylation state. For CHH DMRs, high methylation tends to be the minor state. Thus, it seems that CG DMRs may represent methylation loss and CHH representing methylation gain (if we assume major methylation state is the ancestral state). This figure has been added to the supplemental materials (Figure S2b) and this information is now described briefly in the results.*

(7)By design, most DMRs tagged by SNPs should have a relatively high Minor Epiallele Frequency (MEF). Thus, if they exist, non-tagged DMRs with a relatively high MEF should be good candidates for pure epigenetic variants.

*Response: The reviewer is right to notice that the DMRs in this study have relatively high MEF. We looked at the percentage of DMRs associated with SNPs for DMRs with different MEF (Figure S5b). We found that DMRs with high MEF are more likely to associate with genetic variations, but we do find 40% DMRs with high MEF (0.2-0.5) that don’t associate with genetic variations, even after we consider the SVs. As the reviewer pointed out, these DMRs are good candidates for pure epigenetic variants. We added these in the Discussion part of the manuscript.*

(8)The first sentence of the discussion should be toned down: "Our genome-wide analysis supports a causative role for many of the DMR-gene expression associations that were detected.

*Response: This sentence has been revised as “Our genome-wide analysis favors the possibility that some DMRs are causes of variations in gene expression.”*

(9)The second sentence of the discussion is very naïve. There are now tens if not hundreds of examples in the literature of a positive effect of DNA methylation on gene expression!

*Response: Thanks for pointing this out. We changed the description and now they read as “The negative effect of DNA methylation on gene expression is likely due to a repressive chromatin environment associated with DNA methylation, while the positive effect of DNA methylation is likely attribute to the recruitment by DNA methylation of specific transcription factors to enhance gene expression”.*

(10)"DNA methylation is an ideal candidate". Why ideal, when epistasis, SVs, rare alleles with large effects, etc. are all equally plausible explanations for the missing heritability problem?

*Response: We modified the original description and now they read as “There are examples for which DNA methylation, rather than genetic variation, at specific loci is the cause of a phenotypic change [Quadrana and Colot, Annu Rev Genet. 2016, 50: 467-491].”*

(11)The conclusion should be toned down.

*Response: We modified the conclusion as suggested “In conclusion, DNA methylation was investigated at a population level in maize. Abundant variation in DNA methylation was identified, only some of which is effectively captured by SNP associations. Variations in DNA methylation can separate maize subgroups, associate with differential gene expression and contribute to phenotypic diversity. This study represents the first effort to perform genome-wide association analysis using epigenetic data (DNA methylation) in a crop species. The findings that DMRs not tagged by genetic variation are prevalent and can cause phenotypic variation suggest that DNA methylation is a candidate to explain a portion of the heritability that is not effectively captured by SNPs.”*

Response to Reviewer 2:

*Response: Thanks very much for the positive comments.*

One aspect that is a bit annoying is the correlation between the GWBS sequencing (B73-Mo17) versus the capture assay (B73-Mo17). While the trends are relatively convincing (R2 of 0.76 for CG and 0.70 for CHG) the correlation for CHH is relatively weak (R2 of 0.36), and I wonder whether this might be the reason why the subsequent analyses with the CHH DMRs show trends conflicting with the other contexts. This applies to Fig5 (positively correlated), Figure S7a (R2= 0.34), S7b (negative =1, positive =0.5), S8a (only two points), S8b (R2=0.55 for negative correlation, R2=0.27 for positive correlations).

*Response: This is a great point and we apologize for not being clearer about some of the correlations. Overall, we do generally find lower reproducibility for CHH methylation (for example see information in Li et al., Plant Physiology 2015, 168:1262-1274 for details) than for CG or CHG. However, the measurements of CHH and DMRs can still be robust. In the previous version of the manuscript we had reported on the correlation of capture and WGBS data for all the regions that are included in the assay, including regions that are not DMRs between B73 and Mo17. Thus, for the CHH methylation, the majority of the regions are centered on 0 which results in a relatively poor correlation. In the revised manuscript, we only included regions that are DMRs in either WGBS or sequence capture assays and the correlation values are much higher (R2 = 0.863, 0.857, 0.607 for CG, CHG and CHH, respectively). We believe the positive correlation between CHH and gene expression is not an artifact as this association has been repeatedly detected in our previous analysis and was reported by others as well (for example see the study from Song et al PLoS Genet. 2015, 11(12) :e1005724). The reviewer is right to notice CHH is usually positively associated with gene expression doesn’t exclude the possibility that CHH can be* *negatively associated with gene expression. The direction is region specific and it is currently unclear what determines the direction of association.*

Similarly, I'm not sure that the Fig S5 is really necessary. While the trends are interesting, I'm not sure they had much to the paper. Also, I would suggest that the authors refrain from using the Arabidopsis nomenclature for the maize mutants. It remains unclear whether cmt3 or cmt1 are the homologues of Zmet5 or Zmet2, and there are excellent data sets available from both lead authors for these mutants.

*Response: We agree with the reviewer and deleted Figure S5. Thanks very much for the suggestions on nomenclature. We used the maize gene name in the revised manuscript.*

Finally, I remain a bit confused with the GWAS data correlating gene expression with DMRs. I think this is likely one of the most interesting part of the paper (together with the causal aspect of DNA methylation), but the authors clearly need to better explain their rational for reaching such conclusion.

*Response: We apologize for not being clearer in this section. This was a topic highlighted by the other reviewer as well. We have added additional detail and text for this section in an attempt to clarify the results for this section.*

Also, minor points: There are a significant number of typos in the text and the figure legends. Please, revise as needed.

*Response: We apologize for the errors in the manuscript and figures. We went through the manuscript and figures carefully and attempted to find and correct these issues. Please point out any areas in which there are issues and we will work to resolve them.*

**Second round of review**

**Reviewer 1**

The reviewers have satisfactorily answered the points I raised.

**Reviewer 2**

The authors have answered satisfactorily to most of the comments I made. I would thus recommend accepting the manuscript in its current form.
